# Diet composition of wild columbiform birds: next-generation sequencing of plant and metazoan DNA in faecal samples

**DOI:** 10.1007/s00114-023-01863-8

**Published:** 2023-07-22

**Authors:** Yvonne R. Schumm, Juan F. Masello, Jennifer Vreugdenhil-Rowlands, Dominik Fischer, Klaus Hillerich, Petra Quillfeldt

**Affiliations:** 1 Department of Animal Ecology & Systematics, Justus Liebig University, Heinrich-Buff-Ring 26-32, 35392 Giessen, Germany; 2 Lewestraat 52, 4481 BE Kloetinge, Netherlands; 3 Clinic for Birds, Reptiles, Amphibians and Fish, Veterinary Faculty, Justus Liebig University, Frankfurter Strasse 114, 35392 Giessen, Germany; 4 Present Address: Zoo Wuppertal, Hubertusallee 30, 42117 Wuppertal, Germany; 5 Röntgenstraße 7, 64823 Groß-Umstadt, Germany

**Keywords:** Common Woodpigeon, DNA metabarcoding, European Turtle Dove, High-throughput sequencing, Molecular diet analysis, Stock Dove

## Abstract

Accurate knowledge of a species’ diets is fundamental to understand their ecological requirements. Next-generation sequencing technology has become a powerful and non-invasive tool for diet reconstruction through DNA metabarcoding. Here, we applied those methods on faecal samples of Common Woodpigeons *Columba palumbus*, European Turtle Doves *Streptopelia turtur*, and Stock Doves *C. oenas* to investigate their dietary composition. By applying primer pairs targeting both the ITS2 region of plant nuclear DNA and the mitochondrial COI region of metazoan DNA, we provide a complete picture of the food ingested and estimate the dietary overlap between the columbiform species during the breeding season. Animal DNA was present very rarely, and a diverse range of plants from the class Spermatopsida dominated the diet, with Asteraceae, Brassicaceae, Cucurbitaceae, Fabaceae, and Poaceae as the most frequently represented families. Generally, we detected a variability between species but also amongst individual samples. Plant species already known from previous studies, mainly visual analyses, could be confirmed for our individuals sampled in Germany and the Netherlands. Our molecular approach revealed new plant taxa, e.g. plants of the families Malvaceae for Woodpigeons, Lythraceae for Turtle Doves, and Pinaceae for Stock Doves, not found in previous studies using visual analyses. Although most of the plant species observed were of wild origin, the majority of cultivated plants found were present in higher frequencies of occurrence, suggesting that cultivated food items likely constitute an important part of the diet of the studied species. For Turtle Doves, a comparison with previous studies suggested regional differences, and that food items (historically) considered as important part of their diet, such as Fumitory *Fumaria* sp. and Chickweed *Stellaria media*, were missing in our samples. This indicates that regional variations as well as historic and current data on diet should be considered to plan tailored seed mixtures, which are currently proposed as an important management measure for conservation of the rapidly declining Turtle Dove.

**Supplementary Information:**

The online version contains supplementary material available at 10.1007/s00114-023-01863-8.

## Introduction

Analyses of diet are important to understand the feeding ecology and habitat requirements of animals as well as to manage and protect species (Oehm et al. 2011; Gong et al. 2019). Conventional methods of dietary studies rely on visually identifying diet components during foraging (behavioural observations) or within stomachs, guts, or faeces (morphological classification). These techniques often suffer from misidentification of similar-looking prey items, underrepresentation of soft-bodied or small components, and low taxonomic resolution due to observation distance or digestion stage (Jordan 2005; Oehm et al. 2011; Bowser et al. 2013; Gong et al. 2019). Nowadays, next-generation sequencing (NGS) technology is regularly used as a non-invasive approach for dietary analyses across a variety of animal taxa (e.g. Dunn et al. 2018; Chow et al. 2019; Buglione et al. 2020; Krey et al. 2020) with faeces being the most popular sample type (Alberdi et al. 2018). Some studies have shown that the results of molecular dietary analyses can be helpful in shaping effective, information-based conservation strategies for (endangered) species, e.g. by food resource management (e.g. Ando 2019; Hanson et al. 2021; Zhao et al. 2022).

However, up to now, detailed information about the range and composition of the diets of many free-ranging animals is still limited, and often, only a generalised approximation of the food items consumed is known. Accurate and comprehensive knowledge of the feeding habits and ecology of a species is important to understand its ecological requirements and to evaluate how food availability can affect its population status and to identify key resources for designing management strategies (Wood 1954; Newton 1998; Jordan 2005; Valentini et al. 2008; Gutiérrez-Galán et al. 2017).

From the mid-twentieth century onwards, populations of farmland birds have steeply declined in Europe, partly due to the process of agricultural intensification (Donald et al. 2001; Butler et al. 2010; Reif and Vermouzek 2019). Declines in farmland-associated birds have been linked with reduced numbers of wild plants (Donald et al. 2001; Newton 2004). Agricultural intensification along with changes in farming practise has caused a serious decrease in the abundance and the availability of seeds from wild plants in European farmland areas (Richner et al. 2015; Andreasen et al. 2018; Tarjuelo et al. 2019). Species of Columbiformes native to Europe, including amongst others Common Woodpigeon *Columba palumbus*, European Turtle Dove *Streptopelia turtur*, and Stock Dove *C*. *oenas*, rank amongst the most common and widespread birds in European landscapes, with overlapping occurrence of the different species in habitats such as farmland (Walker 2007; Floigl et al. 2022). Whilst the population trends of Woodpigeons and Stock Doves are moderately increasing, Turtle Doves are rapidly declining across their entire European breeding range (− 33% since 1998; Lormée et al. 2020; PECBMS 2021). It has been suggested that the decline is associated with a reduction in food availability during important periods of the breeding season (Browne and Aebischer 2004; Dunn et al. 2015, 2018; Gutiérrez-Galán and Alonso 2016). This is thought, as the population decline occurred concurrently with decreases in the abundance of many non-cultivated plants in arable habitats, e.g. due to declining fallow land (Sauser et al. 2022), along with a decrease in reproductive output (Calladine et al. 1997; Browne and Aebischer 2004; Dunn et al. 2018). The Turtle Dove is one of the very few long-distance migrant species that are obligate granivorous (Carboneras et al. 2022). The diet of Stock Doves and particularly Woodpigeons include also green plant material, fruits, or invertebrates, especially if seed availability is low (Murton et al. 1964; Möckel 1988; Gutiérrez-Galán et al. 2017; Negrier et al. 2021). The diet of Woodpigeons, considered a granivorous-frugivorous species, has been studied more extensively, particularly in earlier years, likely because they are important game birds and were appraised as a pest of growing crops (Ückermann 1985; Negrier et al. 2021). The diet of Stock Doves seems less intensely studied, although they are also game birds (Romero et al. 2020). In the UK, NGS technology was already used to analyse the diet of different native species of Columbiformes. However, the authors emphasise, that in particular their data, which does originate from other columbiform species than Turtle Doves, should be considered preliminary (Dunn et al. 2018). Generally, most current data on the diet of wild Columbiformes is based on non-molecular, conventional methods and geographically restricted (e.g. Gutiérrez-Galán et al. 2017; Kaouachi et al. 2021; Carboneras et al. 2022).

The present study is aimed at improving our knowledge on the diet composition of three species of Columbiformes (Woodpigeon, Turtle Dove, and Stock Dove) in locations (Germany and the Netherlands) where their feeding ecology was little studied in recent years. NGS technology was used to generate a diet reconstruction through DNA metabarcoding based on faecal samples. Furthermore, we show how the results of the diet reconstruction could help the implementation of management strategies for conserving declining species such as the Turtle Dove.

## Material and methods

### Faecal sample collection and DNA isolation

Faecal samples (*n* = 139, Table 1) were collected from Woodpigeons (*n* = 49), Turtle Doves (*n* = 19), and Stock Doves (*n* = 71) at different sampling sites in Germany and the Netherlands (Fig. S1, Table S1). These sampling sites could not be randomly selected and were rather geographically and unevenly distributed (Fig. S1). They also differed in their habitat composition, e.g. in their proportion of surrounding agricultural areas, which we assessed from land cover data provided by Copernicus Land Monitoring Service (2021; Table S2). The location of sampling sites depended on the presence of the targeted bird species, the applicability of catching techniques, and the likelihood to obtain capture permits, not everywhere granted. Birds were caught using mist nets, trapping cages, and clap nets or in the case of some Stock Doves traps installed to their artificial nest boxes. Faecal samples were collected either opportunistically from the bird during handling or from the inside of clean bird bags within which the birds were temporarily held. Some faecal samples of Woodpigeons were collected as fresh droppings of active nests or roosting sites (*n* = 26) or from transport containers of individuals brought to the clinic for birds by the public (‘Vetmed’, *n* = 19). Some individuals were caught at temporarily baited sites with seeds used to lure individuals (Table S1). Thus, we expected a small amount of baited seeds (Table S3) to be present in the diet of those individuals that were using baited sites (cf. Dunn et al. 2018). Sampled nestlings were at least one week or older to ensure they did not receive crop milk only (Glutz von Blotzheim and Bauer 1987). All faecal samples were stored dark and frozen at − 20 °C.

**Table 1:** Overview of collected faecal samples, separated by age of sampled birds (*Adult*, *Juvenile* = bird already left the nest (fledgling) and is not older than one year and *Nestling* = bird still in nest, but at least one week old). Breeding season is defined as the time period from the 1^st^ of April to the 31^st^ of August

Species	Total number [in breeding season]	Peak in plant PCR [in breeding season]	Peak in metazoa PCR [in breeding season]
Woodpigeon			
Total	49 [29]	32* [24*]	46 [28]
Adult	30 [17]	18 [14]	27 [16]
Juvenile	15 [10]	10 [8]	15 [10]
Nestling	4 [2]	4 [2]	4 [2]
Turtle Dove			
Total	19 [19]	18 [18]	19 [19]
Adult	18 [18]	17 [17]	18 [18]
Juvenile	1 [1]	1 [1]	1 [1]
Stock Dove			
Total	71 [70]	54 [54]	70 [70]
Adult	28 [28]	20 [20]	28 [28]
Juvenile	1 [1]	1 [1]	1 [1]
Nestling	42 [41]	33 [33]	41 [41]

*One sample (RT19_K05) had a peak, but contained no valid MOTU, resulting in 31 [23] faecal samples containing at least one valid plant MOTU

Prior to DNA isolation, 180–200 µg of each sample were weighed. If less material was available, the entire sample was used (minimum: 21 µg). DNA was extracted using the QIAamp ® Fast DNA Stool Kit Mini (QIAGEN GmbH, Germany) with the following modifications to the manufacturer’s instructions: 2–3 bashing beads (ZR Bashing Bead™ 2.0 mm, Zymo Research, USA) were added to ensure proper homogenisation using the Disruptor Genie™ (Scientific Industries SI™). Incubation with Buffer AL and proteinase K was increased from 10 to 30 min.

Two negative extraction controls, i.e., empty Eppendorf tubes, were run along with the faecal samples during isolation and through the entire process. DNA concentration was determined with a NanoDrop2000c UV–Vis Spectrophotometer (NanoDrop Technologies, USA), and samples were diluted to 20 ng/µl if the DNA concentration was higher than 100 ng/µl.

### Construction of sequencing library

A sequencing library (NGS library) was constructed with two consecutive PCR reactions: First, an amplicon PCR followed by an indexing PCR. Initial tests (Supplementary material A1) resulted in the following amplicon PCRs. We used primers UniPlantF and UniPlantR amplifying a 187 to 380 bp region encompassing the second internal transcribed spacer of nuclear ribosomal DNA (ITS2) of plants (Moorhouse-Gann et al. 2018). The primer pair mICOIintF/dgHCO-2198 (Meyer 2003; Leray et al. 2013) was used to amplify a fragment of approx. 300 bp of the highly variable mitochondrial cytochrome* c* oxidase subunit 1 (COI) region of metazoan DNA (Supplementary material A2). All used primers had Illumina overhang adapters attached (P5 for forward primers: 5′-TCGTCGGCAGCGTCAGATGTGTATAAGAGACAG-3′; P7 for reverse primers: 5′-GTCTCGTGGGCTCGGAGATGTGTATAAGAGACAG-3′). PCR runs included PCR grade water as negative control, the negative extraction controls, as well as positive controls (DNA isolated directly from plants or Gastropoda). PCR amplicons were visualised using QIAxcel Advanced (QIAGEN) high-resolution capillary gel electrophoresis.

A 5 µl aliquot of the amplicon PCR products was purified using an Illustra™ ExoproStar 1-Step Kit for enzymatic PCR clean-up (GE Healthcare, UK) according to the manufacturer’s protocol. After this purification, an index PCR was performed in order to individually mark each PCR product with specific Illumina indices added to the P5 and P7 sequencing adapters (Supplementary material A2).

Index PCR products were purified and normalised with a SequalPrep™ Normalization Plate Kit (Thermo Fisher Scientific, USA), and 2 µl of each normalised and individually tagged sample was pooled to finalise the NGS library. In total, 136 and 104 samples were successfully amplified with the metazoan and plant primers, respectively, and sent for sequencing (Table 1, Table S1). The library was sequenced using 250‐bp paired‐end reads on a MiSeq desktop sequencer (Illumina) at SEQ-IT GmbH & Co. KG, Kaiserslautern, Germany.

### Bioinformatics analyses of sequences from faecal samples

To transform the raw Illumina sequence data into a list of MOTUs (molecular operational taxonomic units) with assigned taxonomy, a custom workflow (Masello et al. 2021; for detailed steps see Supplementary material A3) in GALAXY (https://www.computational.bio.uni-giessen.de/galaxy; Afgan et al. 2018) was used.

Subsequently, MOTUs that corresponded to regular fieldwork contaminants in faecal samples (bacteria, soil fungi, and bird DNA) were manually discarded (Kleinschmidt et al. 2019). As short fragments are less likely to contain reliable taxonomic information (Deagle et al. 2009), sequences with a length of less than 100 bp were discarded. Additionally, BLASTn assignment matches of less than 98% were also discarded. MOTUs were assigned to the lowest shared taxonomic level (Kleinschmidt et al. 2019; Table S4). Those that could not be determined at least at family level were excluded.

Further filter steps were performed to obtain reliable data, i.e., avoid contamination and false positives (Crisol-Martínez et al. 2016): MOTUs were accepted only if they contained a minimum of five sequences or accounted for > 1% of the maximum total of hits per columbiform species. For each MOTU, we identified the highest read number within the negative samples and removed this MOTU from any sample where the read number was below this threshold.

### Statistical analysis

All statistical analyses were carried out in R v.4.0.4 (R Core Team 2021). For the comparison between the species, we used a sub-dataset: only faecal samples collected in the breeding season, i.e., where all three species could inhabit the same sites at their European breeding grounds, were used. The breeding season time was defined as the 1^st^ of April to the 31^st^ of August. In addition, samples of nestlings were excluded. This results in a sub-dataset of 75 faecal samples (Woodpigeon *n* = 27, Turtle Dove *n* = 19, Stock Dove *n* = 29; Table 1, Table S1). For dietary overlap analyses, we used the presence or absence data of each MOTU or respective genus or family. The frequency of occurrence ‘FOO’ per single columbiform species was calculated as $\mathrm{FOO}\% = (n/t)*100$, where ‘*n*’ is the number of samples, in which the MOTU was detected, and *t* is the total number of considered samples (Table 2).

**Table 2:** General presence of valid MOTUs (molecular operational taxonomic units) of Spermatopsida in the diet of Common Woodpigeon *Columba palumbus* (WP), European Turtle Dove *Streptopelia turtur* (TD), and Stock Dove *C. oenas* (SD) for the total dataset [● = presence]. Given is the frequency of occurrence for respective MOTUs during the breeding season (FOO_br_%; sub-dataset for samples collected between 1^st^ April and 31^st^ August, excluding samples of nestlings) and the category of each MOTU

Order	Family	MOTU	Common name	Category	Presence [FOO_br_%]		
					WP	TD	SD
Apiales	Apiaceae	*Heracleum* sp.	Hogweeds	Natural		● [5.6]	
	Araliaceae	*Hedera* sp.	Ivies	Natural	● [4.8]		●
Asterales	Asteraceae	*Achillea* sp.	Yarrows	Natural	● [19.0]		
		*Achillea millefolium*	Common yarrow	Natural	● [19.0]		●
		*Artemisia vulgaris*	Common mugwort	Natural	● [9.5]		● [4.8]
		*Bellis perennis*	Common daisy	Natural	● [4.8]		
		*Carthamus tinctorius*	Safflower	Fed			● [4.8]
		*Cichorium* sp.	Chicories	Natural			●
		*Cirsum* sp.	Thistles	Natural		● [11.1]	
		*Crepis capillaris*	Smooth hawksbeard	Natural	● [4.8]	● [5.6]	
		*Dittrichia graveolens*	Stinkwort	Natural			● [9.5]
		*Guizotia abyssinica*	Niger seed	Fed	● [9.5]		
		*Helianthus annuus*	Annual sunflower	Fed	● [23.8]	● [11.1]	● [4.8]
		*Hypochaeris radicata*	Catsear	Natural	● [4.8]		
		*Lactuca* sp.	Lettuce	/	● [9.5]		
		*Scorzoneroides autumnalis*	Autumn hawkbit	Natural	● [9.5]		
		*Senecio inaequidens*	Narrow-leaved ragwort	Natural	●		
		*Sonchus* sp.	Sow thistles	Natural	● [4.8]		
		*Taraxacum* sp.	Dandelions	Natural	● [23.8]	● [11.1]	
		*Tripleurospermum* sp.	Mayweeds	Natural			● [14.3]
Boraginales	Boraginaceae	*Echium vulgare*	Blueweed	Natural		● [5.6]	
Brassicales	Brassicaceae	*Raphanus* sp.	Radishes	Brassica			●
		*Sinapis alba*	White mustard	Brassica		● [5.6]	●
		*Brassica* sp.	Cole crops	Brassica	● [19.0]	● [50.0]	● [66.7]
		*Brassica juncea*	Brown mustard	Brassica		● [22.2]	● [28.6]
		*Brassica napus*	Rapeseed	Brassica	● [9.5]	● [22.2]	● [42.9]
		*Brassica oleracea*	Cabbage	Brassica			● [14.3]
		*Brassica rapa*	Bird rape	Brassica	● [9.5]	● [16.7]	● [42.9]
		*Cardamine hirsuta*	Hairy bittercress	Brassica		● [5.6]	
Caryophyllales	Amaranthaceae	*Chenopodium* sp.	Goosefoots	Natural	● [14.3]	● [16.7]	● [14.3]
	Caryophyllaceae	*Cerastium* sp.	Mouse-ear chickweeds	Natural	● [4.8]		
		*Sagina apetala*	Annual pearlwort	Natural	● [4.8]		
		*Silene* sp.	Campions	Natural	● [4.8]		
		*Silene latifolia*	White campion	Natural	● [4.8]		
		*Silene vulgaris*	Bladder campion	Natural			●
		*Stellaria media*	Chickweed	Natural			● [4.8]
Cucurbitales	Cucurbitaceae	*Cucumis* sp.		Cultivated	● [38.1]		● [19.0]
		*Cucurbita* sp.	Gourd	Cultivated	● [61.9]	● [27.8]	● [47.6]
		*Cucurbita pepo*	Field pumpkin	Cultivated	● [38.1]	● [16.7]	● [33.3]
Dipsacales	Adoxaceae	*Sambucus nigra*	Elder	Tree	● [9.5]		
Ericales	Balsaminaceae	*Impatiens* sp.	Snapweeds	natural			●
		*Impatiens parviflora*	Small balsam	Natural			●
Fabales	Fabaceae	*Glycine max*	Soya bean	Cultivated	● [9.5]	● [11.1]	
		*Lotus* sp.	Bird’s-foot trefoils	Natural	● [4.8]		
		*Pisum sativum*	Pea	Cultivated			● [19.0]
		*Robinia* sp.	Locusts	Tree		● [11.1]	
		*Trifolium pratense*	Red clover	Natural	● [4.8]		
		*Trifolium repens*	White clover	Natural	● [9.5]		
		*Vicia* sp.	Vetches	/			● [52.4]
		*Vicia hirsuta*	Hairy vetch	Cultivated			● [33.3]
		*Vicia lathyroides*	Spring vetch	Natural			● [19.0]
		*Vicia sativa*	Common vetch	Cultivated			● [47.6]
		*Vicia sepium*	Bush vetch	Cultivated			●
		*Vicia tetrasperma*	Smooth vetch	Natural			● [9.5]
Fagales	Betulaceae	*Betula* sp.	Birches	Tree	● [9.5]	● [11.1]	
		*Carpinus* sp.	Hornbeams	Tree	● [4.8]		
	Fagaceae	*Fagus* sp.	Beeches	Tree	● [14.3]		●
	Juglandaceae	*Juglans regia*	Walnut	Tree	● [4.8]		● [4.8]
Gentianales	Rubiaceae	*Galium* sp.	Bedstraws	Natural	● [4.8]		
Lamiales	Plantaginaceae	*Hippuris* sp.	Mare’s tails	Natural			● [4.8]
		*Plantago lanceolata*	Buckhorn plantain	Natural	● [19.0]		● [4.8]
		*Veronica chamaedrys*	Cat’s eyes	Natural			● [4.8]
Liliales	Liliaceae	*Lilium* sp.	Lilies	/	● [4.8]		
Malpighiales	Euphorbiaceae	*Euphorbia helioscopia*	Sun spurge	Natural		● [11.1]	● [9.5]
		*Mercurialis annua*	Annual mercury	Natural			● [14.3]
	Linaceae	*Linum* sp.	Flax plants	Cultivated		● [5.6]	
Malvales	Malvaceae	*Tilia* sp.	Linden	Tree	● [9.5]		
		*Tilia platyphyllos*	Large-leaved linden	Tree	● [4.8]		
Myrtales	Lythraceae	*Lythrum salicaria*	Purple loosestrife	Natural		● [5.6]	
	Onagraceae	*Epilobium* sp.	Willowherbs	Natural		● [5.6]	● [4.8]
		*Oenothera* sp.	Evening primroses	Natural			● [14.3]
Pinales	Pinaceae	*Picea* sp.	Spruces	Tree	● [9.5]		● [14.3]
		*Pinus* sp.	Pines	Tree		● [5.6]	
		*Pinus sylvestris*	European red pine	Tree	● [14.3]	● [5.6]	● [4.8]
Poales	Cyperaceae	*Carex* sp.	Sedges	Natural	● [19.0]	● [16.7]	● [28.6]
	Poaceae	*Poaceae*	Grasses	/	● [95.2]	● [94.4]	● [90.5]
		*Agrostis* sp.	Bentgrasses	Natural	● [14.3]		●
		*Alopecurus myosuroides*	Black-grass	Natural			● [4.8]
		*Alopecurus pratensis*	Meadow foxtail	Natural	● [19.0]	● [5.6]	
		*Arrhenatherum* sp.	Oatgrasses	Natural	● [19.0]	● [11.1]	
		*Arrhenatherum elatius*	False oat-grass	Natural	● [19.0]	● [11.1]	
		*Avena* sp.	Oats	/	● [14.3]	● [5.6]	● [9.5]
		*Dactylis glomerata*	Cock’s-foot	Natural	● [4.8]		● [9.5]
		*Elymus* sp.	Couch grasses	Natural			●
		*Festuca* sp.	Fescues	Natural	● [14.3]		
		*Holcus* sp.	Soft-grasses	Natural		● [11.1]	
		*Hordeum vulgare*	Barley	Cultivated	● [28.6]	● [33.3]	● [14.3]
		*Lolium* sp.	Ryegrasses	Natural	● [23.8]		● [4.8]
		*Lolium perenne*	Perennial ryegrass	Natural	● [23.8]		● [4.8]
		*Molinia caerulea*	Purple moorgrass	Natural	● [23.8]		● [9.5]
		*Panicum miliaceum*	Proso millet	Fed	● [23.8]	● [50.0]	● [28.6]
		*Phalaris* sp.		Natural	● [14.3]		
		*Poa* sp.	Meadow-grasses	Natural	● [19.0]	● [11.1]	● [4.8]
		*Poa trivialis*	Rough bluegrass	Natural	● [14.3]		
		*Secale cereale*	Rye	Cultivated	● [4.8]	● [16.7]	● [14.3]
		*Setaria* sp.	Bristle grasses	Fed	● [9.5]	● [11.1]	● [4.8]
		*Trisetum flavescens*	Yellow oatgrass	Cultivated	● [4.8]		
		*Triticum* sp.	Wheat	Cultivated	● [57.1]	● [66.7]	● [76.2]
		*Triticum aestivum*	Common wheat	Cultivated	● [9.5]	● [16.7]	● [28.6]
		*Triticum dicoccon*	Emmer wheat	Cultivated	● [9.5]		
		*Triticum* sp.*elta*	Dinkel wheat	Cultivated			● [4.8]
		*Zea mays*	Maize	Cultivated	● [23.8]	● [16.7]	● [19.0]
Ranunculales	Ranunculaceae	*Ranunculus* sp.	Buttercups	Natural		● [27.8]	
Rosales	Cannabaceae	*Cannabis sativa*	Hemp	Fed	● [33.3]	● [27.8]	● [38.1]
	Elaeagnaceae	*Hippophae rhamnoides*	Sea-buckthorn	/			● [4.8]
	Rosaceae	*Amelanchier* sp.	Shadbushes	Tree	● [9.5]		
		*Potentilla* sp.	Cinquefoils	Natural	● [4.8]		
		*Prunus* sp.		Tree	● [23.8]		● [4.8]
		*Prunus avium*	Bird cherry	Tree	● [4.8]		
		*Rosa* sp.	Roses	Natural	●	● [11.1]	
		*Rubus* sp.		Natural	● [4.8]	● [22.2]	●
	Urticaceae	*Urtica dioica*	Common nettle	Natural	● [14.3]	● [16.7]	● [14.3]
Sapindales	Sapindaceae	*Acer* sp.	Maples	Tree	● [19.0]		● [9.5]
		*Acer platanoides*	Norway maple	Tree	● [4.8]		
		*Acer pseudoplatanus*	Sycamore	Tree	● [9.5]		
Saxifragales	Crassulaceae	*Sedum* sp.	Stonecrops	Natural	●		
Solanales	Convolvulaceae	*Convolvulus arvensis*	Field bindweed	Natural	● [4.8]		
	Solanaceae	*Solanum lycopersicum*	Tomato	Cultivated	●		● [14.3]

Since the data are qualitative data, we tested for differences in diet species composition at family and genus level with permutation tests in the R package ‘VEGAN’ (Oksanen et al. 2009). Non-metric Multidimensional Scaling (NMDS, function *metaMDS*) was used to visualise species differences in diet compositions. NMDS uses rank orders to collapse information from multiple dimensions into usually two dimensions to facilitate visualisation as well as interpretation and is generally considered the most robust unconstrained ordination method in community ecology (Faith et al. 1987; Minchin 1987). The function *metaMDS* allowed us to investigate the agreement between the two-dimension configuration and the original configuration through a stress parameter (if the stress value < 0.1 the agreement is very good, < 0.2 is a good representation). For this analysis at family level samples containing only a single plant family (*n* = 5) were discarded. Stress values in the present tests were < 0.26 at family and < 0.17 at genus level. We performed Permutational Multivariate Analysis of Variance Using Distance Matrices (PERMANOVA) with the function *adonis* and checked for the multivariate homogeneity of group dispersions (variances) with the function *betadisper*.

To assess the dietary overlap of each species pair according to the presence/absence data at family and genus level of valid MOTUs, we calculated Pianka’s measure of overlap *O*_*jk*_ (Pianka 1986) in the R package ‘SPAA’ (Zhang 2016) using the *niche.overlap* function.

To evaluate differences between the avian species in the consumption of different plant species in the form of relative proportions, we categorised the MOTUs in broad categories following the concept proposed by Dunn et al. (2018). The dietary components (MOTUs) were classified in the following five broad categories according to their likely source (Table 2, Table S4): ‘fed’ (seeds likely to be offered at feeders), ‘cultivated’ (crop plants as well as those widely cultivated as components of seed mixes sown to provide seed for wild birds), ‘natural’ (wild plant species), ‘tree’, and ‘brassica’ (all MOTUs of the family Brassicaceae). ‘Brassica’ was considered a separate category as the family of Brassicaceae includes plants used to provision birds, as well as cultivated and several naturally occurring wild species (cf. Dunn et al. 2018). If a species and respective genus occurred both as separate MOTUs in one species, e.g. *Achillea* sp. and *Achillea millefolium*, they were combined for categorisation.

## Results

### Diet composition—metazoan DNA

Apart from the consumption of a few insects (9 samples, Table S4), only one valid metazoan prey MOTU was present in the faecal sample of one Stock Dove. This was the DNA of a Common Earthworm *Lumbricus terrestris* (Table S4). Due to the low presence of animal prey in our samples, the further statistical evaluation refers only to the plant components found in the faecal samples.

### Diet composition—plants

Of all faecal samples successfully amplified with the plant primers (*n* = 103; Woodpigeon *n* = 31, Turtle Dove *n* = 18, Stock Dove *n* = 54; Table 1, Table S1), at least one valid MOTU was found in every sample with an average of 9.5 ± 5.8 MOTUs per sample (maximum: 33 MOTUs in one sample). A total of 118 MOTUs were found, with 54.2% of MOTUs determined at species level, 44.9% at genus level, and 0.8% at family level. All MOTUs belonged to the class Spermatopsida, distributed amongst 23 orders and 34 families (Table 2).

### Diet differences amongst species of Columbiformes

**Fig. 1:**
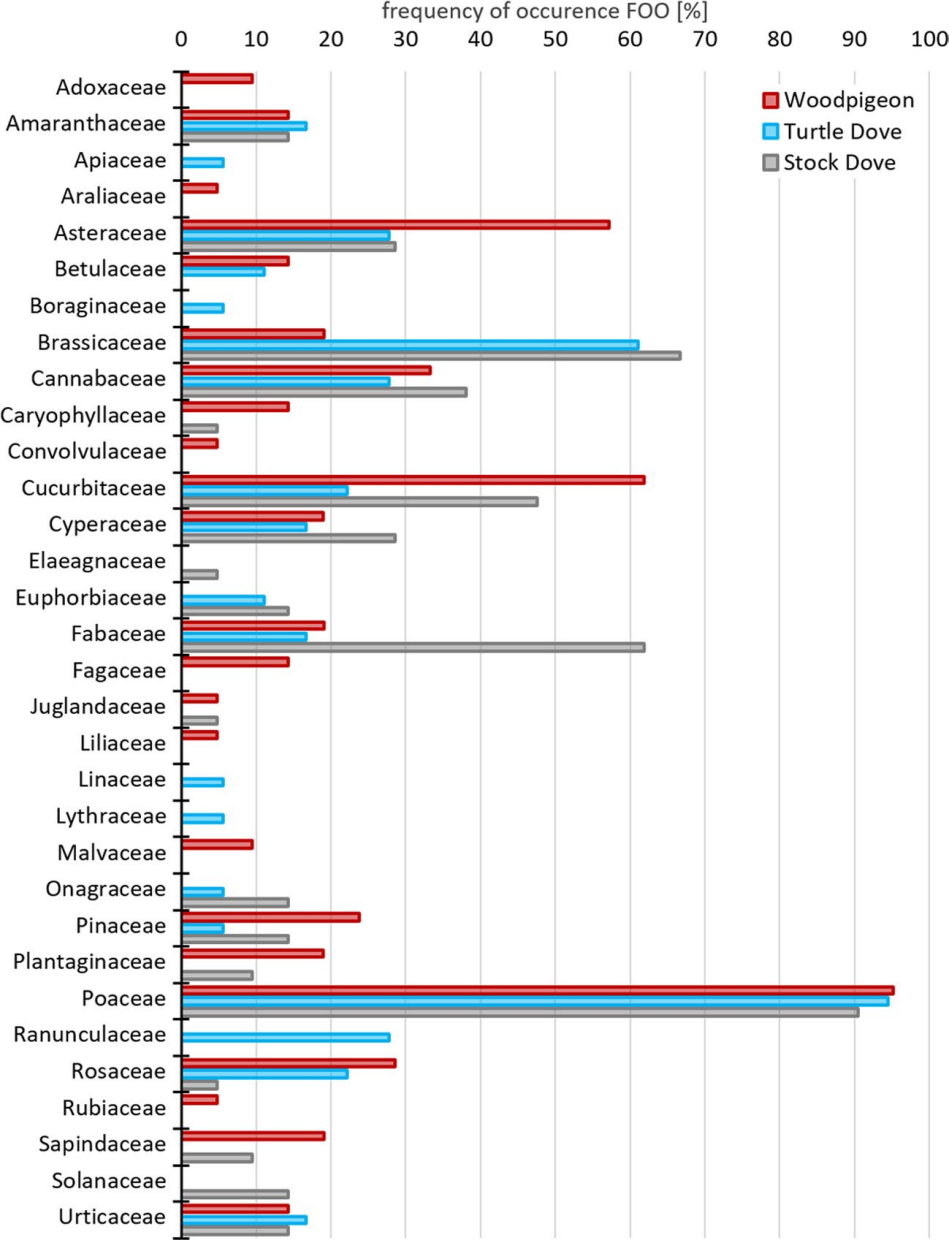
Diet composition of Common Woodpigeon *Columba palumbus*, European Turtle Dove *Streptopelia turtur*, and Stock Dove *C. oenas*. Summary of plant families found in faecal samples, collected during the breeding season (1^st^ of April to 31^st^ of August) represented as the frequency of occurrence (FOO%). Faecal samples of nestlings were excluded

Within the sub-dataset (only breeding season and nestlings excluded), a total of 110 MOTUs were found with 19.1% of MOTUs present in all three species of Columbiformes, 20.9% in two species, and 60.0% in only one bird species (Table 2). Most MOTUs were found in Woodpigeons (75 MOTUs), followed by Stock Doves (54) and Turtle Doves (44). The most represented plant families, occurring with a FOO_br_% (frequency of occurrence within the sub-dataset) of at least 50% in one species, were Asteraceae, Brassicaceae, Cucurbitaceae, Fabaceae, and Poaceae (Fig. 1).

**Fig. 2:**
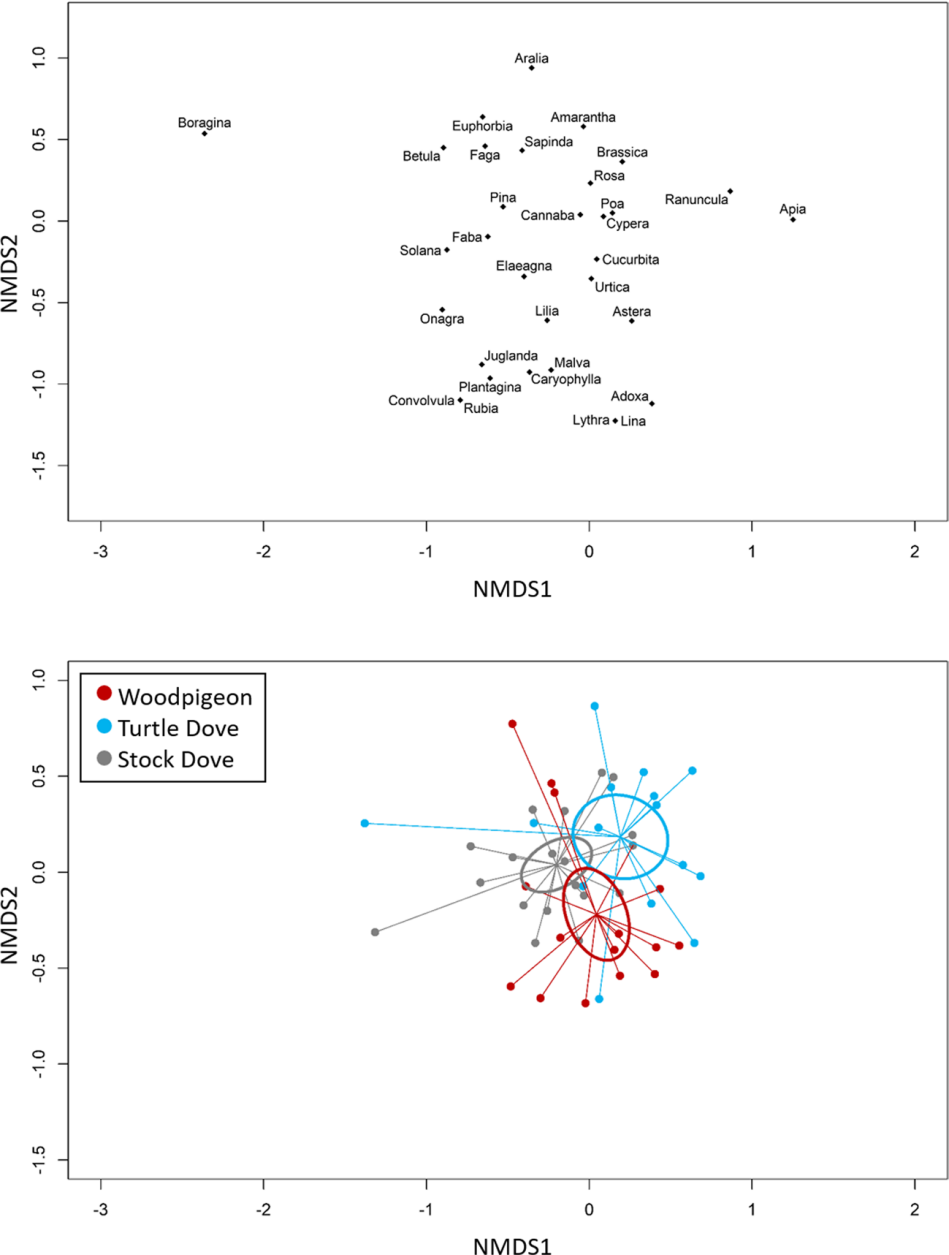
Differences in the diet composition at plant family level in three columbiform species (Common Woodpigeon *Columba palumbus*; European Turtle Dove *Streptopelia turtur*; Stock Dove *C. oenas*), using Non-metric Multidimensional Scaling (NMDS, function *metaMDS* in the R package ‘VEGAN’). Depicted are on the top the distribution of the plant families (the word ending ‘-ceae’ was removed to avoid overlapping between the labels and improve readability) and the distribution of samples and 95% confidence ellipses (bottom)

Overall, the community analysis showed that the diet composition differed between the columbiform species at plant family level (Fig. 2) as indicated by permutation tests (permutation test for differences: *F*_52,2_ = 3.8, *p* < 0.001). However, the difference in species explained only 12.9% of the overall variation (*R*^2^ = 0.129). Likewise, the result at genus level (Fig. S2) pointed out differences between the species’ diet composition (permutation test for differences: *F*_57,2_ = 3.1, *p* < 0.001), though this difference also explained a rather small proportion (9.7%) of the overall variation (*R*^2^ = 0.097).

According to Pianka’s measure of overlap at family level Woodpigeon and Stock Dove showed the highest dietary overlap (*O*_*jk*_ = 0.718), followed by Stock Dove and Turtle Dove (*O*_*jk*_ = 0.684). Woodpigeon and Turtle Dove had the least plant families in common (*O*_*jk*_ = 0.574). Also at genus level, Woodpigeon and Stock Dove showed the highest dietary overlap (*O*_*jk*_ = 0.549), albeit the similarities were lower than at family level. The overlap between Turtle Dove and Woodpigeon (*O*_*jk*_ = 0.499) as well as Turtle Dove and Stock Dove (*O*_*jk*_ = 0.495) was relatively equal.

**Fig. 3:**
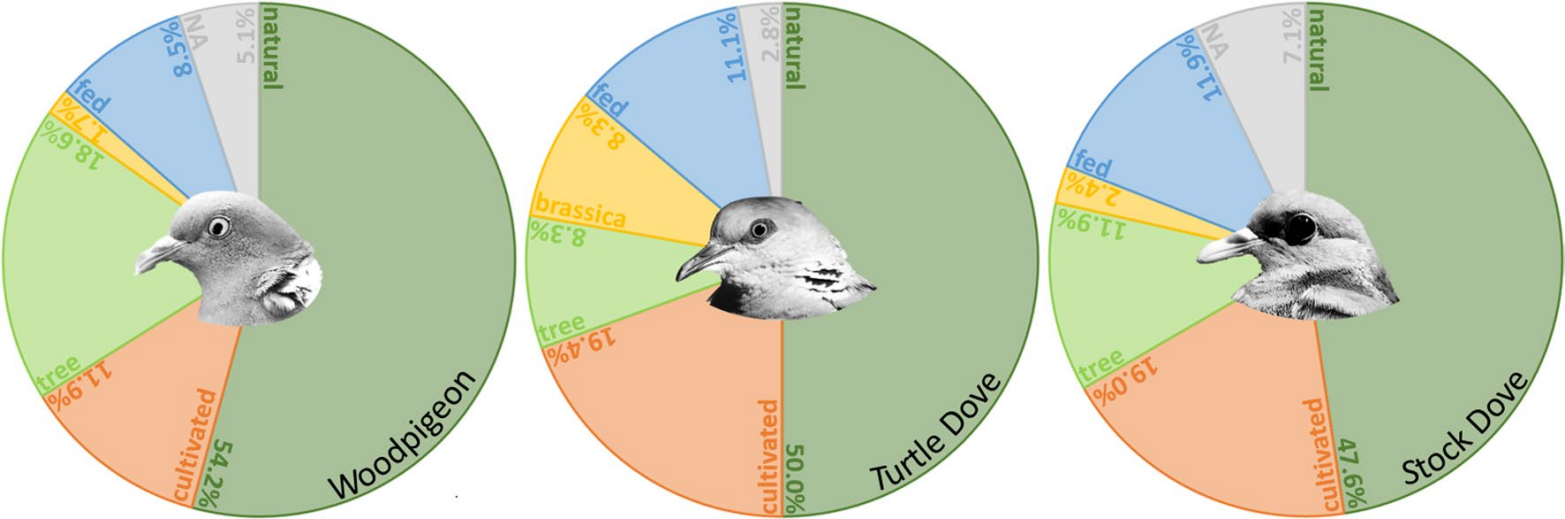
Relative proportion of dietary component categories during the breeding season (nestlings excluded) of three columbiform species (Common Woodpigeon *Columba palumbus*, European Turtle Dove *Streptopelia turtur*, and Stock Dove *C. oenas*). The categories reflect the likely source of the dietary component (MOTU, Table S4). Proportion is given as percent [%] based on the presence/absence data of MOTUs per species (not based on a quantitative assessment of the proportion of consumed plants, e.g. from sequence read numbers (read abundance)). ‘NA’ indicates that the MOTU could not be assigned clearly to a category

Considering all 118 MOTUs from the complete dataset, most MOTUs were assigned to the category ‘natural’ (53.4%), followed by ‘cultivated’ (15.3%), and ‘tree’ (14.4%). The remaining MOTUs were categorised as ‘brassica’ (6.8%) or ‘fed’ (5.1%). Some MOTUs (5.1%) could not be clearly assigned (‘NA’) to one of the categories (Table S4). Comparing the three species (sub-dataset), also most MOTUs were assigned to the category ‘natural’ (Woodpigeon = 54.2%, Turtle Dove = 50.0%, and Stock Dove = 47.6%), followed by ‘cultivated’ for Turtle Dove (19.4%) and Stock Dove (19.0%) and ‘tree’ for Woodpigeon (18.6%; Fig. 3). None of the proportion of categories varied significantly between the columbiform species (pairwise *t*-test all *χ*^2^ ≤ 2.4, df = 1, *p* ≥ 0.118).

## Discussion

### Diet reconstruction based on NGS technology and comparison with previous studies

#### Animal constituents

The DNA data from faecal samples of the columbiform species show a diverse range of taxa, dominated mainly by plant constituents, whilst animal prey was present very rarely. In line with our study, most other studies found no or only little numbers of animal material. In a review, the proportion of invertebrate components in the diet of all three species of Columbiformes was below 5% (Holland et al. 2006).

Murton et al. (1964) reported the intake of cocoons of Earthworms for Stock Doves. We found Earthworm DNA in the faeces of one Stock Dove. However, with the applied method, we cannot determine the development stage (cocoon, larvae or imago). In Woodpigeons, animal constituents were observed with small volumes and low frequency (Ó hUallachain and Dunne 2013; Gutiérrez-Galán et al. 2017; Negrier et al. 2021) or were completely absent (Jimenez et al. 1994; Kaouachi et al. 2021). Animal prey was present in very few samples of Turtle Doves in Spain or completely absent in other years (Jimenez et al. 1992; Gutiérrez-Galán and Alonso 2016).

A few insect species were detected in the faecal samples (Table S4). Insects and Crustacea have occasionally been found in previous studies, e.g. Coccoidea, larvae, and cocoons of Lepidoptera in Woodpigeons (Murton et al. 1964; Glutz von Blotzheim and Bauer 1987; Ó hUallachain and Dunne 2013) and Cecidomyiidae larvae or Coleoptera in Stock Doves (Möckel 1988). However, these animals were likely not consumed on purpose but taken ‘accidentally’ whilst eating plant components or during plumage care.

#### Plant constituents

Results obtained in the diet of Columbiformes showed a wide diversity in consumed plants. The applied molecular DNA metabarcoding approach detected a larger number of plant families than former analyses based on visual or observational identification of food items (Table S5-7).

The Woodpigeon is regarded as opportunistic feeder, feeding on various food items and switching to alternative species when preferred ones are unavailable, which can lead to a pronounced seasonal variation (Gutiérrez-Galán et al. 2017; Kaouachi et al. 2021). This rather generalist feeding is also reflected in our results, as Woodpigeon samples contained the highest number of plant families (*n* = 23 in the sub-dataset; Fig. 1). Most of the plant families detected in our samples were already described as part of Woodpigeon diet. Interestingly, we found plants of the families Crassulaceae, Juglandaceae, Liliaceae, Malvaceae, and Sapindaceae, which were not mentioned in previous studies (Table S5). Some MOTUs found for Woodpigeons in our study might differ from previous studies, as many studies concentrated on sampling in rural and agricultural areas, whereas most of our Woodpigeon samples originated from (sub-)urban habitats, i.e., 49% of Woodpigeon samples were collected from the sample site ‘Giessen’, which is dominated by the land cover category ‘artificial surfaces’ as placed in the medium-sized city of Giessen (Copernicus Land Monitoring Service 2021; Table S1, Table S2). Once a typical and exclusive woodland species, Woodpigeons colonised cities of Western and Central Europe since the early nineteenth century (Fey et al. 2015). Urban areas typically contain novel food items, such as non-native species and intentionally (e.g. bird feeders) or unintentionally provisioned food (e.g. garbage in landfills). Therefore, many wildlife species shift their diets to use these ‘novel’ food resources (Murray et al. 2018).

Some examples for the food items that were most likely found in urban areas solely are the MOTUs *Amelanchier* sp. (ornamental shrub), *Sedum* sp. (ornamental garden plant; roof covering in green roofs), and *Lilium* sp.(ornamental plant). Other MOTUs likely originate from food provided in bird feeders (Table 1, category ‘fed’): relatively frequently found in Woodpigeon faecal samples were e.g. Sunflower *Helianthus annuus*, Niger Seed *Guizotia abyssinica*, and Proso Millet *Panicum miliaceum*. Provided seeds in urban areas, e.g. wheat, maize, or millet, have probably also contributed to the high FOO% of Poaceae (96.8%) in Woodpigeons, but it also is known that individuals from (sub-)urban areas move out to agricultural areas to feed upon farmland there (Slater 2001; Table S2). Overall, the diet of the Woodpigeon fits into the known pattern with some peculiarities in the diet of individuals from urban areas. Here, it would be interesting to compare the diet of the Woodpigeon and Eurasian Collared Dove *S*. *decaocto*, as these two species are likely to co-exist in (sub-)urban areas (Floigl et al. 2022). Unfortunately, we were not able to catch any Collared Dove in our sampling sites.

The Turtle Dove is considered a obligate granivorous bird (Fisher et al. 2018). Glutz von Blotzheim and Bauer (1987) name seeds of Polygonaceae, Papaveraceae, Brassicaceae, Asteraceae, Poaceae, Pinaceae, Faboideae, and *Chenopodium* sp. to constitute the main diet on the breeding grounds. Fumitory *Fumaria* sp. historically formed the mainstay of Turtle Dove diet in the UK (Murton et al. 1964). Individuals sampled in the UK also commonly ate other natural plants, e.g. *S. media*, Scarlet Pimpernel *Anagallis arvensis*, Cock’s-foot *Dactylis glomerata*, *Poa* sp., Geraniaceae, and Amaranthaceae (Murton et al. 1964; Dunn et al. 2018; Fisher et al. 2018). Whilst Poaceae (FOO = 94.4%), Brassicaceae (61.1%), Asteraceae (27.8%), Faboideae (16.7%), *Chenopodium* sp. (16.7%), and Pinaceae (5.6%) occurred in our Turtle Dove samples, Polygonaceae and Papaveraceae were not detected (Table 2). In the UK, it was shown that the feeding ecology of the Turtle Dove changed significantly from non-cultivated, natural arable plants, primarily weed seeds, to mainly cultivated plants, such as rape and wheat, from the 1960s to the late 1990s (Browne and Aebischer 2003). The authors of this study reported that changes in agricultural practises have reduced or removed many of the feeding opportunities, such as hayfields or clover leys, available at their study location, and that concurrently, increased use of herbicides and fertilisers as well as more efficient screening procedures have reduced weed abundance and diversity. The observed dietary shift might be therefore largely influenced by the spatial and temporal availability of certain diet items, particularly of the natural plants. Unfortunately, studies assessing specifically the availability of seeds are very scarce (Carboneras et al. 2022).

Our results also reflect the dietary shift from wild plants to cultivated ones. On the one hand, MOTUs categorised as ‘natural’, except for *Ranunculus s*p. (27.8%) and *Rubus* sp. (22.2%), occurred with FOO lower than 20%, whilst cultivated ones reached higher FOO (*Triticum* sp. = 66.7%; *Brassica* sp. = 50.0%, including *B*. *napus* with 22.2%). On the other hand, we did not find some historically important food items, particularly *Fumaria* sp. and *S. media*, even though they generally grow in Germany and the Netherlands (Sparrius et al. 2014; Metzing et al. 2018). However, our sampling sites and surrounding areas were not specifically surveyed for the presence of these plant species. Similar to our results, these wild plants, classified as important in Turtle Dove diet, in particular in the UK, were also absent in other regions (Romania and Slovakia: Glutz von Blotzheim and Bauer 1987; Russia: Murton et al. 1965; Spain: Gutiérrez-Galán and Alonso 2016). The comparison with previous studies shows that only the plant family Poaceae was present in Turtle Dove diet in all the represented European countries (Table S6). To our knowledge, the families Betulaceae, including the MOTU *Betula* sp., Cyperaceae (MOTU: *Carex* sp.), and Lythraceae (MOTU: *Lythrum salicaria*) were so far not mentioned as part of Turtle Dove diet (Table S6). Seeds provided at bird feeders, such as Hemp *Cannabis sativa*, Niger *Guizotia abyssinica*, and *Sorghum* sp., were recently found in Turtle Dove diet in the UK (Dunn et al. 2018), possibly indicating a further range addition in their dietary spectrum. In our Turtle Dove samples of these three MOTUs, only *C*. *sativa* was found. *G*. *abyssinica* was found in our sample set, but only in Woodpigeon samples (Table 2). Generally, Dunn et al. (2018) warn that the addition of wild bird seed mixes to the dietary spectrum of Turtle Doves may have negative consequences, such as the increased exposure to parasites such as the flagellate *Trichomonas gallinae* at shared water and food sources.

In a recent review, it was shown that Turtle Doves were found to feed mainly on annual ruderal plants. However, a large number of seed types were reported across European breeding grounds, underlining the wide variety of seeds consumed by this species (Carboneras et al. 2022). The observed regional dietary differences may be due to climatic and biogeographical differences as well as variation in habitat, e.g. agricultural landscape vs forest, and occurrence and availability of certain plant species (Gutiérrez-Galán and Alonso 2016; Mansouri et al. 2019).

For Stock Doves, we detected 22 plant families (Table S7). Previously, seeds and fruits of plants from the families Poaceae, Fabaceae, Brassicaceae, Polygonaceae and Caryophyllaceae were described as the most important food items (Glutz von Blotzheim and Bauer 1987), whereby especially seeds of wild and cultivated vetches *Vicia* sp. (Fabaceae) comprise a major part (Murton et al. 1965; Möckel 1988). In line with this, four of the five aforementioned plant families were present in Stock Doves sampled in our study (Fig. 1). The important proportion of vetches for Stock Dove diet is also supported by our data. *Vicia* DNA could be traced in 55.6% of all Stock Dove samples with the Hairy Vetch *Vicia hirsuta* being the most frequent (Table 2), whereas *Vicia* DNA was not found in Woodpigeon or Turtle Dove samples. Nine plant families have not been mentioned as being part of Stock Dove diet in previous studies (Table S7).

### Dietary composition differences between species

The degree of dietary overlap between the studied columbiform species pairs was slightly lower than that observed by Dunn et al. (2018) with Pianka’s measure, ranging from 0.7 to 0.9 compared to 0.5 to 0.7 in our study. Dietary overlap between the species suggests that some resources are shared and the species might compete for food, assuming that the shared resources are limited. However, it has been suggested that the related columbiform species select different feeding sites, occupy different ecological niches, or utilise superabundant supplies if taking the same food items, indicating rather little or no competition between them (Murton et al. 1964; Jimenez et al. 1994). The permutation tests indicated significant variance in diet composition amongst the species (Fig. 2, Fig. S2). However, both at plant family and genus level, the differences amongst species explained only a rather small proportion of the overall variation (12.9% and 9.7%, respectively). This implies a rather pronounced variability within species, which is also supported by the rather strongly varying number of MOTUs detected per sample (1 to 33). With the use of DNA metabarcoding, we cannot distinguish which part of the plant was eaten and the different species may feed on different parts of the same plant species; e.g. Woodpigeons eat the young leaves of Brassicaceae, whereas Turtle Doves feed on Brassica seeds. This can result in the degree of dietary overlap being overestimated. In general, the study design could be improved by including only samples of individuals from all wild columbiform species (optimally also Collared Dove), which co-exist in the same location to ascertain more accurately their dietary overlap or potential competition on certain food items (cf. Benghedier et al. 2020; Squalli et al. 2022). This approach would also prevent possible impacts of the local area, e.g. varying habitat composition surrounding the sampling sites, as it was present in our study (see Table S2 for an assessment of the surrounding habitat composition of the respective capture sites). Thus, future studies should survey data of the local area around sampling sites (e.g. habitat composition, plant species abundance, and availability of seeds) where possible. Another limitation of the method is that only the presence/absence data of food items are obtained, i.e., an evaluation of taxonomic richness, and thus, quantitative assessment of the proportion of consumed plants (i.e. taxon-specific proportions) is not possible and results for species comparison should be considered preliminary (cf. Dunn et al. 2018). Even if the metabarcoding technique possesses the ability for quantitative assessment, the current understanding of the factors affecting the quantitative performance of DNA metabarcoding is still limited and uncertainties remain. Thus, additional research is required before metabarcoding can be confidently utilised for quantitative applications such as the quantitative assessment of diet component proportions (Lamb et al. 2019; Ando et al. 2020; Stapleton et al. 2022; Shelton et al. 2023). Therefore, even though in our study most MOTUs were assigned to the category ‘natural’ for all three species (Fig. 3), it cannot be assumed that these proportionally form the main part of the diet. Based on previous, non-molecular studies, seeds and plant material of ‘cultivated’ species are expected to constitute the main fraction of the diet. In Woodpigeons sampled in Spain, 97.6% in volume corresponded to cultivated plants (Jimenez et al. 1994). Wheat and rape seeds averaged 61% of the seeds eaten by Turtle Doves in the UK (Browne and Aebischer 2003). In Stock Doves, Wheat and Barley made up 80–90% of the diet in April (Möckel 1988). Faecal metabarcoding was used in our study to identify dietary items and taxonomic richness of dietary composition in the three species of Columbiformes. Future developments and improvements of the methodology, as well as validation studies (e.g. Verkuil et al. 2022), would also make possible to determine the taxon-specific proportions of dietary items. This would also allow to answer ecological questions, such as how do species separate their trophic niches in space and time or what are the consequences of (seasonal) food availability and consumption for food-webs, more accurately than is possible with a pure listing of consumed species (Verkuil et al. 2022).

### Application of results for management strategies for conservation

Given the Turtle Dove’s specialised diet on seeds, the development of an extensive, seed-provisioning option, i.e. increased food availability, is considered vital for management actions to conserve the species, whereby options to enhance food availability should favour the provision of wild seeds rather than cultivated seeds (Dunn et al. 2015; Carboneras et al. 2022). For instance, it was shown that the condition of Turtle Dove nestlings fed with cultivated seeds was poorer than that of those fed with wild seeds (Dunn et al. 2018). Most existing options, e.g. agri‐environment schemes (AES) or agri-environmental policies (AEP), seem suboptimal in providing accessible food for Turtle Doves (Dunn et al. 2015). A tailored sown mix, based on plant species known to be present in Turtle Dove diet historically, has been devised by an RSPB/Natural England project aiming to provide optimal foraging conditions. However, even though the sown plots provided more seeds compared to control plots, sown plots developed a too dense vegetation structure to attract foraging Turtle Doves. Therefore, modifications for the tailored sown mix were recommended (Dunn et al. 2015).

The results of our study combined with results of other previous studies, and knowledge from existing tailored sown mixtures (e.g. Dunn et al. 2015, 2021), were used to set up an agri-environmental scheme called ‘*Turteltauben Brache*’ in Hesse, Germany. This was done in the framework of the hessian HALM-programme (HALM is short for: Hessian Programme for Agri-environmental and Landscape Management Measures), aimed at creating suitable foraging sites for Turtle Doves with a sufficient abundance of seeds. For detailed recommended sown mix, see Table S8, and for further management instructions, see Schumm et al. (2022). However, there is no experience so far how these created foraging sites will develop according to the recommended multi-year management. Future studies must investigate whether these sites produce abundant seeds, which can successfully be exploited by Turtle Doves.

The preservation of (existing) fallow areas can also contribute to the protection of Turtle Doves, as these can be a suitable seed-rich foraging habitat. They were shown to have a positive effect on Turtle Dove abundance, as well as on the abundance of other farmland bird species (Vickery et al. 2004; Dunn and Morris 2012; Dunn et al. 2021; Sauser et al. 2022; Staggenborg and Anthes 2022). A study conducted in Germany comparing agri-environment measures showed that some plant species only occur in fallows and that the presence of fallows has an overall positive effect on plant species richness and abundance in arable habitats (Wietzke et al. 2020). Typical plant species growing on (arable) fallows, which we found in Turtle Dove samples, are e.g. Blueweed *Echium vulgare*, Common Nettle *Urtica dioica*, Goosefoots *Chenopodium* sp. (Table 2).

Updated and improved knowledge of the seeds included in Turtle Dove diet will help to define, optimise and carry out tailored management options, such as tailored sowings or management of seminatural habitats (e.g. fallows or grasslands) as well as optimise feeding during rehabilitation and possible ex situ conservation projects, particularly as data on the diet is fragmentary and limited (Mansouri et al. 2019). Our results and their comparison with previous studies highlight the presence of regional differences in Turtle Dove diet composition (see also Carboneras et al. 2022) and that some plant species (historically) considered important food items in some regions, might not be the major part of Turtle Dove diet in other regions. Further studies should focus on identifying regional dietary differences as they might play an important role in planning tailored seed mixes. It is probably advisable to plan the composition of seed mixtures according to locally preferred and known wild plant species in order to achieve the best possible acceptance of the managed foraging and feeding areas by the Turtle Doves. This conclusion as well as the use of metabarcoding as a non-invasive approach for diet analysis and observation of diet shifts may also be relevant for the management and conservation of other declining farmland-associated bird species as many share the loss of food resources in terms of seeds and also invertebrates (Bowler et al. 2019; Tallamy and Shriver 2021). For instance, it can be useful to leave in the sites the plants created with the sown seed mixtures standing over the winter to promote structural diversity and cover (for resident and overwintering species), as well as the presence of insects with reproductive cycles spanning the winter time. Generally, it was shown that the sowing of seed mixtures or flowering mixtures (creation of flowering strips) can enhance the species richness of insects (e.g. Toivonen et al. 2016; Buhk et al. 2018); thus, they can increase the food resource availability for insectivore, granivore, and mixed feeding avian species, e.g. Common Linnet *Linaria cannabina*, Common Reed Bunting *Emberiza schoeniclus*, Grey Partridge *Perdix perdix*, or Meadow Pipit *Anthus pratensis* (Ronnenberg et al. 2016; Redhead et al. 2018; Bowler et al. 2019; Tallamy and Shriver 2021; Staggenborg and Anthes 2022). However, the detailed mechanisms of the impact of agricultural intensification on population decline may vary between species. More detailed knowledge on species-specific diet and dietary requirements could help to understand the mechanisms of decline and help mitigating the ongoing declining population trends. This is particularly important considering a meta-analysis that showed how some farmland species of high conservation concern profited most strongly from targeted management programmes (Staggenborg and Anthes 2022). However, Staggenborg and Anthes (2022) pointed out that in their meta-analysis they found relevant associations between targeted and non-targeted programmes and bird abundance for only five of nine avian farmland species, indicating that the management and conservation programmes need to be further optimised in order to meet the respective (ecological) requirements of target species and should be adjusted to local circumstances.

## Supplementary Information

Below is the link to the electronic supplementary material.

Supplementary file1 (DOCX 759 KB)
