# Supplementary material for: Diet composition of wild columbiform birds: next-generation sequencing of plant and metazoan DNA in faecal samples
Source: Naturwissenschaften. 2023 Jul 22;110(4):38. doi: 10.1007/s00114-023-01863-8 (PMC10363069; doi:10.1007/s00114-023-01863-8)
Supplement: Supplementary file 1 — Supplementary file1 (DOCX 759 KB) [file 114_2023_1863_MOESM1_ESM.docx]

**Supporting information**

**Appendix A**

**Diet composition of wild columbiform birds: next-generation sequencing of plant and metazoan DNA in faecal samples**

*The Science of Nature*

Yvonne R. Schumm^*^, Juan F. Masello, Jennifer Vreugdenhil-Rowlands, Dominik Fischer, Klaus Hillerich, Petra Quillfeldt

* Corresponding author: Yvonne.R.Schumm@bio.uni-giessen.de, Department of Animal Ecology & Systematics, Justus Liebig University, Heinrich-Buff-Ring 26-32, 35392 Giessen, Germany

**Supplementary material A1**. Initial tests for amplicon PCR

In order to choose the most suitable primer pair and combination of primers and polymerase initial tests were carried out with a subset of the samples:

**1)** Comparison of polymerases Multiplex PCR Mastermix (QIAGEN GmbH, Germany) and Q5 Hot Start High Fidelity DNA Polymerase (New England BioLabs Inc, USA) for the primer pair used for amplification of plant material (UniPlantF and UniPlantR, Moorhouse-Gann et al. 2018) with a subset of 43 samples. More samples with clear peaks during adapter PCR and more valid MOTUs (molecular operational taxonomic units) were determined when Q5 DNA Polymerase was applied (28 vs 17 and 83 vs 49, respectively).

**2)** Comparison of primer pairs applied to detect animal prey material: primer pair BilSSU1100_F and BilSSU1300_R (Jarman et al. 2004) vs mICOIintF and dgHCO-2198 (Meyer 2003; Leray et al. 2013) with a subset of 17 samples. While for both primer pairs all samples rendered a positive peak, with the mlCOIintF/dgHCO-2198 primers MOTUs could be determined to a lower taxonomic level (species vs family).

References:

Jarman SN, Deagle BE, Gales NJ (2004) Group-specific polymerase chain reaction for DNA-based analysis of species diversity and identity in dietary samples. Mol Ecol 13:1313–1322. https://doi.org/10.1111/j.1365-294X.2004.02109.x

Leray M, Yang JY, Meyer CP, et al. (2013) A new versatile primer set targeting a short fragment of the mitochondrial COI region for metabarcoding metazoan diversity: application for characterizing coral reef fish gut contents. Front Zool 10:34. https://doi.org/10.1186/1742-9994-10-34

Meyer CP (2003) Molecular systematics of cowries (Gastropoda: Cypraeidae) and diversification patterns in the tropics. Biol J Linn Soc 79:401–459. https://doi.org/10.1046/j.1095-8312.2003.00197.x

Moorhouse-Gann R, Dunn J, de Vere N, Goder M, Cole N, Hipperson H, Symondson WOC (2018) New universal ITS2 primers for high‐resolution herbivory analyses using DNA metabarcoding in both tropical and temperate zones. Sci Rep 8:8542. https://doi.org/10.1038/s41598-018-26648-2

**Supplementary material A2**. Amplicon and index PCR setups and cycling conditions

UniPlantF and UniPlantR amplicon PCR

The 12.5 μl PCR reaction volumes for the amplicon PCR consisted of 2.5 μl Q5 Reaction Buffer, 0.15 µl Q5 Hot Start High Fidelity DNA Polymerase, 0.5 μl of each primer (10 µM), 0.05 µl BSA (20 mg/ml), 0.5 µl dNTPs (10 µM of each dNTP), 6.3 µl nuclease-free water and 2 µl DNA template. Thermal cycling conditions were 95°C for 15 min, followed by 40 cycles of 95°C for 30 s, 56°C for 30 s, and 72°C for 60 s, followed by a final extension at 72°C for 10 min.

mICOIintF and dgHCO-2198 amplicon PCR

The 20 µl amplicon PCR setup consisted of 10 µl Multiplex PCR Mastermix, 0.4 µl of each primer (10 µM), 0.1 µl BSA, 7.1 µl nuclease-free water and 2 µl template DNA. Cycling conditions were set to 95°C for 15 min, followed by 35 cycles of 94°C for 30 s, 48°C for 90 s, 72°C for 90 s and a final extension at 72 °C for 10 min.

Index PCR

The 30 µl index PCR reactions contained 6 µl cleaned amplicon PCR product, 2.1 µl of each of the two respective indices (10 µM), 12.3 µl nuclease-free water and for plant primers 6 µl Q5 Reaction Buffer, 0.3 µl Q5 Polymerase and 1.2 µl dNTPs and for metazoan primers 7.5 µl Multiplex PCR Mastermix. Thermal cycling conditions were 95°C for 1 min, followed by 7 cycles of 95°C for 10 s, 56°C for 30 s, and 72°C for 30 s; and a final extension at 72°C for 5 min.

**Supplementary material A3**. GALAXY workflow

A custom workflow in GALAXY was used to transform the raw Illumina sequence data into a list of MOTUs (molecular operational taxonomic units) with assigned taxonomy. The workflow included: pre-filtering for index quality (Wright and Vetsigian 2016) to avoid cross-talk among multiplexed samples (e.g. plant dataset: 417,775 paired‐end reads with index filter vs 537,558 without applied index filter), assessing sequence quality with FASTQC (http://www.bioinformatics.babraham.ac.uk/projects/fastqc); adapter and quality trimming of the paired-end reads with TRIMMOMATIC (Bolger et al. 2014); merging of the overlapping paired-end read pairs using FLASH (Magoč and Salzberg 2011); transforming sequence files to FASTA with the FASTX-Toolkit (http://hannonlab.cshl.edu/fastx_toolkit/); and extracting amplicons in MOTHUR (Schloss et al. 2009). USEARCH (Edgar 2010) was used to remove identical replicates (dereplicate; derep_fulllength), to detect and to remove chimeric sequences (uchime_denovo) and to cluster sequences into MOTUs. Using the BLASTn algorithm MOTU sequences were matched to reference sequences in the NCBI GenBank nucleotide database, using a cut-off of 90% minimum sequence identity and a maximum e-value of 0.00001 (Altschul et al. 1990).

References:

Altschul SF, Gish W, Miller W, Myers EW, Lipman DJ (1990) Basic local alignment search tool. J Mol Biol 215:403–410. https://doi.org/10.1016/S0022-2836(05)80360-2

Bolger AM, Lohse M, Usadel B (2014) Trimmomatic: A flexible trimmer for Illumina sequence data. Bioinformatics 30:2114–2120. https://doi.org/10.1093/bioinformatics/btu170

Edgar RC (2010) Search and clustering orders of magnitude faster than BLAST’. Bioinformatics 26:2460–2461. https://doi.org/10.1093/bioinformatics/btq461

Magoč T, Salzberg SL (2011) FLASH: Fast length adjustment of short reads to improve genome assemblies. Bioinformatics 27:2957–2963. https://doi.org/10.1093/bioinformatics/btr507

Schloss PD, Westcott SL, Ryabin T, et al. (2009) Introducing mothur: Open-source, platform-independent, community-supported software for describing and comparing microbial communities. Appl Environ Microbiol 75:7537–7541. https://doi.org/10.1128/AEM.01541-09

Wright ES, Vetsigian KH (2016) Quality filtering of Illumina index reads mitigates sample cross-talk. BMC genomics 17:1–7. https://doi.org/10.1186/s12864-016-3217-x

**Table S1**. Collected faecal samples from three species of the order Columbiformes (WP = Common Woodpigeon *Columba palumbus*, TD = European Turtle Dove *Streptopelia turtur*, SD = Stock Dove *C. oenas*) at different sampling sites in Germany and the Netherlands from the years 2013 to 2020 and results of amplicon PCR amplifications of plant and metazoan DNA. Samples used for species comparison (within breeding season and no nestling) are given in bold.

| **Sample ID** | **Species** | **Sample site** | **Baited site** | **Age^1^** | **Collection Date**  **[dd.mm.yyyy]** | **Peak in plant PCR^2^** | **Peak in metazoan PCR^2^** |
| --- | --- | --- | --- | --- | --- | --- | --- |
| **TT18_K01** | TD | Weilbacher Kiesgruben | Yes | adult | 21.06.2018 | Yes | Yes |
| **TT18_K02** | TD | Weilbacher Kiesgruben | Yes | adult | 25.06.2018 | Yes | Yes |
| **BB1** | TD | Lieberoser Heide | Yes | adult | 01.07.2018 | Yes | Yes |
| **TT19_K01** | TD | Lieberoser Heide | Yes | adult | 25.06.2019 | Yes | Yes |
| **TT19_K02** | TD | Lieberoser Heide | Yes | adult | 25.06.2019 | Yes | Yes |
| **TT19_K03** | TD | Lieberoser Heide | Yes | adult | 26.06.2019 | Yes | Yes |
| **TT19_K04** | TD | Lieberoser Heide | Yes | adult | 26.06.2019 | Yes | Yes |
| **TT19_K05** | TD | Lieberoser Heide | Yes | adult | 26.06.2019 | Yes | Yes |
| **TT_NLK01** | TD | Zak van Zuid-Beveland | Yes | adult | 13.05.2019 | Yes | Yes |
| **TT_NLK02** | TD | Zak van Zuid-Beveland | Yes | adult | 01.07.2019 | Yes | Yes |
| **TT_NLK03** | TD | Zak van Zuid-Beveland | Yes | juvenile | 01.07.2019 | Yes | Yes |
| **TT20_K01** | TD | Cleeberg | Yes | adult | 05.06.2020 | Yes | Yes |
| **TT20_K02** | TD | Cleeberg | Yes | adult | 05.06.2020 | Yes | Yes |
| **TT20_K03** | TD | Cleeberg | Yes | adult | 07.06.2020 | Yes | Yes |
| **TT20_K04** | TD | Hungen-Villingen | Yes | adult | 08.06.2020 | Yes | Yes |
| **TT20_HEL_K01** | TD | Helgoland | No | adult | 08.05.2020 | Yes | Yes |
| **TT20_NL_K01** | TD | Zak van Zuid-Beveland | Yes | adult | 30.05.2020 | No | Yes |
| **TT20_NL_K02** | TD | Zak van Zuid-Beveland | Yes | adult | 13.06.2020 | Yes | Yes |
| **TT20_NL_K03** | TD | Zak van Zuid-Beveland | Yes | adult | 13.06.2020 | Yes | Yes |
| HT13_K10 | SD | Groß-Umstadt | No | nestling | 01.06.2013 | Yes | Yes |
| **HT13_K21** | SD | Groß-Umstadt | No | adult | 01.06.2013 | Yes | Yes |
| HT13_K26 | SD | Groß-Umstadt | No | nestling | 17.07.2013 | Yes | Yes |
| **HT14_K1** | SD | Groß-Umstadt | No | adult | 26.04.2014 | No | Yes |
| HT14_K2 | SD | Groß-Umstadt | No | nestling | 26.04.2014 | No | Yes |
| **HT14_K3** | SD | Groß-Umstadt | No | adult | 26.04.2014 | No | Yes |
| HT14_K5 | SD | Groß-Umstadt | No | nestling | 26.04.2014 | Yes | Yes |
| HT14_K6 | SD | Groß-Umstadt | No | nestling | 26.04.2014 | Yes | Yes |
| **HT14_K7** | SD | Groß-Umstadt | No | adult | 26.04.2014 | No | Yes |
| **HT14_K8** | SD | Groß-Umstadt | No | adult | 26.04.2014 | No | Yes |
| HT14_K10 | SD | Groß-Umstadt | No | nestling | 26.04.2014 | No | Yes |
| HT14_K12 | SD | Groß-Umstadt | No | nestling | 26.04.2014 | Yes | Yes |
| HT14_K13 | SD | Groß-Umstadt | No | nestling | 26.04.2014 | No | Yes |
| HT14_K17 | SD | Groß-Umstadt | No | nestling | 15.05.2014 | No | Yes |
| HT14_K22 | SD | Groß-Umstadt | No | nestling | 15.05.2014 | No | Yes |
| **HT14_K25** | SD | Groß-Umstadt | No | adult | 15.05.2014 | Yes | Yes |
| HT14_K26/27 | SD | Groß-Umstadt | No | nestling | 15.05.2014 | Yes | Yes |
| **HT15_K14.08.2015** | SD | Lausitz | Yes | adult | 14.08.2015 | No | Yes |
| **HT15_KLau2** | SD | Lausitz | Yes | adult | 14.08.2015 | No | Yes |
| HT18_K01 | SD | Groß-Umstadt | No | nestling | 17.05.2018 | Yes | Yes |
| HT18_K02 | SD | Groß-Umstadt | No | nestling | 17.05.2018 | Yes | Yes |
| HT18_K03 | SD | Groß-Umstadt | No | nestling | 17.05.2018 | No | Yes |
| HT18_K04 | SD | Groß-Umstadt | No | nestling | 17.05.2018 | No | Yes |
| HT18_K05 | SD | Groß-Umstadt | No | nestling | 17.05.2018 | Yes | Yes |
| HT18_K06 | SD | Groß-Umstadt | No | nestling | 17.05.2018 | Yes | Yes |
| **HT18_K07** | SD | Groß-Umstadt | No | adult | 22.06.2018 | Yes | Yes |
| HT18_K08 | SD | Groß-Umstadt | No | nestling | 22.06.2018 | Yes | Yes |
| HT18_K09 | SD | Groß-Umstadt | No | nestling | 22.06.2018 | Yes | Yes |
| **HT18_K10** | SD | Eichkopf | Yes | adult | 02.07.2018 | Yes | Yes |
| **HT18_K11** | SD | Groß-Umstadt | No | adult | 19.07.2018 | Yes | Yes |
| **HT18_K12** | SD | Groß-Umstadt | No | adult | 19.07.2018 | Yes | Yes |
| **HT18_K13** | SD | Groß-Umstadt | No | adult | 19.07.2018 | Yes | Yes |
| **Sample ID** | **Species** | **Sample site** | **Baited site** | **Age^1^** | **Collection Date**  **[dd.mm.yyyy]** | **Peak in plant PCR^2^** | **Peak in metazoan PCR^2^** |
| HT18_K14 | SD | Groß-Umstadt | No | nestling | 19.07.2018 | Yes | Yes |
| HT18_K15 | SD | Groß-Umstadt | No | nestling | 02.08.2018 | Yes | Yes |
| HT18_K16 | SD | Groß-Umstadt | No | nestling | 20.08.2018 | Yes | Yes |
| HT18_K17 | SD | Groß-Umstadt | No | nestling | 20.08.2018 | Yes | Yes |
| **HT19_K01** | SD | Weilbacher Kiesgruben | Yes | adult | 17.06.2019 | No | Yes |
| **HT19_K02** | SD | Weilbacher Kiesgruben | Yes | adult | 17.06.2019 | Yes | Yes |
| **HT19_K03** | SD | Weilbacher Kiesgruben | Yes | adult | 17.06.2019 | Yes | Yes |
| **HT19_K04** | SD | Weilbacher Kiesgruben | Yes | adult | 18.06.2019 | Yes | Yes |
| **HT19_K05** | SD | Weilbacher Kiesgruben | Yes | adult | 18.06.2019 | Yes | Yes |
| **HT19_K06** | SD | Eichkopf | Yes | adult | 18.06.2019 | Yes | Yes |
| **HT19_K07** | SD | Weilbacher Kiesgruben | Yes | adult | 19.06.2019 | Yes | Yes |
| **HT19_K08** | SD | Weilbacher Kiesgruben | Yes | adult | 02.07.2019 | Yes | Yes |
| **HT19_K09** | SD | Weilbacher Kiesgruben | Yes | juvenile | 08.07.2019 | Yes | Yes |
| HT19_K10 | SD | Groß-Umstadt | No | nestling | 08.07.2019 | Yes | Yes |
| HT19_K11 | SD | Groß-Umstadt | No | nestling | 08.08.2019 | Yes | Yes |
| HT19_K12 | SD | Groß-Umstadt | No | nestling | 08.08.2019 | Yes | Yes |
| **HT19_K13** | SD | Groß-Umstadt | No | adult | 08.08.2019 | Yes | Yes |
| **HT19_K14** | SD | Groß-Umstadt | No | adult | 08.08.2019 | Yes | Yes |
| **HT19_K15** | SD | Groß-Umstadt | No | adult | 16.08.2019 | No | Yes |
| **HT19_K16** | SD | Groß-Umstadt | No | adult | 16.08.2019 | Yes | Yes |
| **HT19_K17** | SD | Groß-Umstadt | No | adult | 16.08.2019 | Yes | Yes |
| HT19_K18 | SD | Groß-Umstadt | No | nestling | 22.08.2019 | Yes | Yes |
| HT19_K19 | SD | Groß-Umstadt | No | nestling | 22.08.2019 | Yes | Yes |
| HT19_K20 | SD | Groß-Umstadt | No | nestling | 22.08.2019 | Yes | Yes |
| HT19_K21 | SD | Groß-Umstadt | No | nestling | 22.08.2019 | Yes | Yes |
| HT19_K22 | SD | Groß-Umstadt | No | nestling | 23.08.2019 | Yes | Yes |
| **HT19_K23** | SD | Groß-Umstadt | No | adult | 23.08.2019 | Yes | Yes |
| **HT19_K24** | SD | Groß-Umstadt | No | adult | 23.08.2019 | Yes | Yes |
| HT19_KK01 | SD | Zeulenroda | No | nestling | 22.04.2019 | Yes | Yes |
| HT19_KK02 | SD | Zeulenroda | No | nestling | 27.04.2019 | Yes | Yes |
| HT19_KK03 | SD | Zeulenroda | No | nestling | 12.05.2019 | Yes | Yes |
| HT19_KK04 | SD | Zeulenroda | No | nestling | 06.06.2019 | No | Yes |
| HT19_KK05 | SD | Zeulenroda | No | nestling | 18.07.2019 | Yes | Yes |
| HT19_KK06 | SD | Zeulenroda | No | nestling | 30.08.2019 | Yes | Yes |
| HT19_KK07 | SD | Zeulenroda | No | nestling | 30.08.2019 | Yes | Yes |
| HT19_KK08 | SD | Zeulenroda | No | nestling | 30.08.2019 | Yes | Yes |
| HT19_KK09 | SD | Zeulenroda | No | nestling | 30.08.2019 | Yes | Yes |
| HT19_KK10 | SD | Zeulenroda | No | nestling | 30.08.2019 | Yes | Yes |
| HT19_KK11 | SD | Zeulenroda | No | nestling | 02.10.2019 | No | No |
| **RT18_K01** | WP | Giessen | No | adult | 08.06.2018 | Yes | Yes |
| **RT18_K02** | WP | Giessen | No | adult | 13.06.2018 | Yes | Yes |
| **RT18_K03** | WP | Giessen | Yes | adult | 29.06.2018 | Yes | Yes |
| RT18_K04 | WP | Giessen | Yes | adult | 13.12.2018 | No | Yes |
| RT19_K01 | WP | Giessen | Yes | adult | 17.01.2019 | No | Yes |
| RT19_K02 | WP | Giessen | No | adult | 27.02.2019 | Yes | Yes |
| RT19_K03 | WP | Giessen | Yes | adult | 07.03.2019 | Yes | Yes |
| RT19_K04 | WP | Giessen | No | adult | 14.03.2019 | No | No |
| **RT19_K05** | WP | Giessen | No | adult | 03.05.2019 | Yes* | Yes |
| **RT19_K06** | WP | Giessen | No | adult | 09.05.2019 | Yes | Yes |
| **RT19_K07** | WP | Giessen | No | adult | 14.06.2019 | Yes | Yes |
| **RT19_K08** | WP | Caldern | Yes | adult | 18.06.2019 | No | Yes |
| **Vet_K01** | WP | Vetmed | No | juvenile | 19.06.2019 | Yes | Yes |
| **Vet_K02** | WP | Vetmed | No | juvenile | 21.06.2019 | No | Yes |
| **Vet_K03** | WP | Vetmed | No | adult | 04.07.2019 | Yes | Yes |
| **RT19_K09** | WP | Giessen | No | adult | 09.07.2019 | Yes | Yes |
| **RT19_K10** | WP | Giessen | No | adult | 10.07.2019 | Yes | Yes |
| **Vet_K04** | WP | Vetmed | No | juvenile | 11.07.2019 | Yes | Yes |
| **Sample ID** | **Species** | **Sample site** | **Baited site** | **Age^1^** | **Collection Date**  **[dd.mm.yyyy]** | **Peak in plant PCR^2^** | **Peak in metazoan PCR^2^** |
| **Vet_K05** | WP | Vetmed | No | juvenile | 11.07.2019 | Yes | Yes |
| **Vet_K06** | WP | Vetmed | No | juvenile | 22.07.2019 | Yes | Yes |
| **Vet_K07** | WP | Vetmed | No | juvenile | 22.07.2019 | Yes | Yes |
| **RT19_K11** | WP | Giessen | No | adult | 22.07.2019 | Yes | Yes |
| **Vet_K08** | WP | Vetmed | No | juvenile | 24.07.2019 | Yes | Yes |
| **Vet_K09** | WP | Vetmed | No | juvenile | 24.07.2019 | No | Yes |
| **Vet_K10** | WP | Vetmed | No | juvenile | 31.07.2019 | Yes | Yes |
| **RT19_K12** | WP | Giessen | No | adult | 07.08.2019 | Yes | Yes |
| **Vet_K11** | WP | Vetmed | No | juvenile | 09.08.2019 | Yes | Yes |
| **Vet_K12** | WP | Vetmed | No | adult | 20.08.2019 | Yes | Yes |
| RT19_K13 | WP | Giessen | No | nestling | 28.08.2019 | Yes | Yes |
| RT19_K14 | WP | Giessen | No | nestling | 30.08.2019 | Yes | Yes |
| Vet_K13 | WP | Vetmed | No | adult | 02.09.2019 | Yes | Yes |
| RT19_K15 | WP | Giessen | No | nestling | 03.09.2019 | Yes | Yes |
| RT19_K16 | WP | Giessen | No | nestling | 06.09.2019 | Yes | Yes |
| Vet_K14 | WP | Vetmed | No | juvenile | 19.09.2019 | No | Yes |
| Vet_K15 | WP | Vetmed | No | adult | 19.09.2019 | No | Yes |
| Vet_K16 | WP | Vetmed | No | juvenile | 19.09.2019 | Yes | Yes |
| Vet_K17 | WP | Vetmed | No | juvenile | 20.09.2019 | Yes | Yes |
| Vet_K18 | WP | Vetmed | No | juvenile | 26.09.2019 | No | Yes |
| RT19_K18 | WP | Giessen | No | adult | 29.09.2019 | Yes | Yes |
| Vet_K19 | WP | Vetmed | No | juvenile | 03.10.2019 | No | Yes |
| RT20_K01 | WP | Wilhelmshaven | No | adult | 28.02.2020 | No | Yes |
| RT20_K02 | WP | Wilhelmshaven | No | adult | 28.02.2020 | No | No |
| RT20_K03 | WP | Wilhelmshaven | No | adult | 28.02.2020 | No | Yes |
| RT20_K04 | WP | Wilhelmshaven | No | adult | 28.02.2020 | No | Yes |
| RT20_K05 | WP | Wilhelmshaven | No | adult | 28.02.2020 | No | Yes |
| **RT20_K06** | WP | Giessen | No | adult | 24.04.2020 | Yes | Yes |
| **RT20_K07** | WP | Giessen | No | adult | 10.06.2020 | No | Yes |
| **RT20_K08** | WP | Giessen | No | adult | 18.06.2020 | Yes | Yes |
| **RT20_K09** | WP | Giessen | No | adult | 18.06.2020 | No | Yes |

^1^ age of sampled birds. Juvenile = bird already left the nest (fledgling) and is not older than one year.

Nestling = bird still in nest, but at least one week old. NA = age is unknown, e.g. samples collected

underneath roosting trees without seeing the single bird

^2^ peak at the expected band height during the amplicon PCR

* had a peak, but contained no valid MOTU

**Table S2.** Proportional occurrence [%] of Corine Land Cover classes (Copernicus Land Monitoring Service 2021) in the areas (2.5 km radius circles) around the sample sites. For definitions of Land Cover classes, please see https://land.copernicus.eu/user-corner/technical-library/corine-land-cover-nomenclature-guidelines/html/. Information is given which species was sampled at which site in general (please see Table S1 to see which specific sample destined from which sample site; WP = Common Woodpigeon *Columba palumbus*, TD = European Turtle Dove *Streptopelia turtur*, SD = Stock Dove C. oenas). See Figure S1 for a map of the sample sites.

| Land Cover class | Sample site [sampled species] | | | | | | | | | | | | |
| --- | --- | --- | --- | --- | --- | --- | --- | --- | --- | --- | --- | --- | --- |
|  | Cleeberg [TD] | Helgoland [TD] | Hungen-Villingen [TD] | Lieberoser Heide [TD] | Zak van Zuid-Beveland [TD] | Weilbacher Kiesgruben [TD, SD] | Giessen [WP] | Caldern [WP] | Wilhemshaven [WP] | Eichkopf [SD] | Groß-Umstadt [SD] | Lausitz [SD] | Zeulenroda [SD] |
| **Artificial Surfaces** | **6** | **2** | **11** | **0** | **0** | **19** | **59** | **7** | **12** | **4** | **10** | **20** | **0** |
| Continuous urban fabric | 0 | 0 | 0 | 0 | 0 | 2 | 8 | 0 | 0 | 0 | 2 | 0 | 0 |
| Discontinuous urban fabric | 4 | 2 | 10 | 0 | 0 | 9 | 23 | 6 | 8 | 4 | 8 | 18 | 0 |
| Industrial or commercial units | 0 | 0 | 1 | 0 | 0 | 4 | 24 | 1 | 3 | 0 | 0 | 0 | 0 |
| Road and rail networks and associated land | 0 | 0 | 0 | 0 | 0 | 0 | 3 | 0 | 0 | 0 | 0 | 0 | 0 |
| Mineral extraction sites | 0 | 0 | 0 | 0 | 0 | 3 | 0 | 0 | 0 | 0 | 0 | 0 | 0 |
| Green urban areas | 0 | 0 | 0 | 0 | 0 | 0 | 0 | 0 | 0 | 0 | 0 | 2 | 0 |
| Sport and leisure facilities | 2 | 0 | 0 | 0 | 0 | 1 | 1 | 0 | 0 | 0 | 0 | 0 | 0 |
| **Agricultural areas** | **38** | **0** | **47** | **12** | **100** | **74** | **14** | **50** | **86** | **22** | **49** | **66** | **70** |
| Non-irrigated arable land | 12 | 0 | 35 | 12 | 63 | 61 | 6 | 37 | 14 | 11 | 33 | 26 | 58 |
| Vineyards | 0 | 0 | 0 | 0 | 0 | 0 | 0 | 0 | 0 | 0 | 4 | 0 | 0 |
| Fruit trees and berry plantations | 0 | 0 | 0 | 0 | 4 | 2 | 0 | 0 | 0 | 0 | 0 | 0 | 0 |
| Pastures | 26 | 0 | 9 | 0 | 17 | 11 | 8 | 13 | 72 | 11 | 10 | 40 | 9 |
| Complex cultivation patterns | 0 | 0 | 1 | 0 | 16 | 0 | 0 | 0 | 0 | 0 | 2 | 0 | 0 |
| Land principally occupied by agriculture with significant areas of natural vegetation | 0 | 0 | 2 | 0 | 0 | 0 | 0 | 0 | 0 | 0 | 0 | 0 | 3 |
| **Forest and seminatural areas** | **55** | **7** | **41** | **89** | **0** | **6** | **27** | **44** | **0** | **74** | **42** | **11** | **22** |
| Broad-leaved forest | 48 | 0 | 41 | 0 | 0 | 6 | 16 | 43 | 0 | 65 | 25 | 0 | 0 |
| Coniferous forest | 2 | 0 | 0 | 58 | 0 | 0 | 10 | 1 | 0 | 6 | 10 | 10 | 22 |
| Mixed forest | 5 | 0 | 0 | 3 | 0 | 0 | 1 | 0 | 0 | 3 | 7 | 1 | 0 |
| Natural grasslands | 0 | 4 | 0 | 0 | 0 | 0 | 0 | 0 | 0 | 0 | 0 | 0 | 0 |
| Moors and heathland | 0 | 2 | 0 | 26 | 0 | 0 | 0 | 0 | 0 | 0 | 0 | 0 | 0 |
| Transitional woodland-shrub | 0 | 0 | 0 | 2 | 0 | 0 | 0 | 0 | 0 | 0 | 0 | 0 | 0 |
| Beaches - dunes - sands | 0 | 1 | 0 | 0 | 0 | 0 | 0 | 0 | 0 | 0 | 0 | 0 | 0 |
| **Water bodies** | **0** | **91** | **0** | **0** | **0** | **1** | **0** | **0** | **2** | **0** | **0** | **2** | **8** |
| Water courses | 0 | 0 | 0 | 0 | 0 | 1 | 0 | 0 | 0 | 0 | 0 | 0 | 0 |
| Water bodies | 0 | 0 | 0 | 0 | 0 | 0 | 0 | 0 | 2 | 0 | 0 | 2 | 8 |
| Sea and ocean | 0 | 91 | 0 | 0 | 0 | 0 | 0 | 0 | 0 | 0 | 0 | 0 | 0 |

**Table S3**. Seed mixes used at baited sites in Germany and Netherlands to attract columbiform species

| **Country** | **Product name (manufacturer)** | **Plant species** |
| --- | --- | --- |
| Germany | Organic 6-grain mixture (Biogewinner, Nienhagen, Germany) | wheat (*Tricticum sp*, 39%), rye (*Secale cereale*, 18%), oat (*Avena sp*, 18%), barley (*Hordeum vulgare*, 10%), millet (10%), buckwheat (*Fagopyrum esculentum*, 10%) |
|  | Organic birdseeds (Biohof Lex, Bockhorn, Germany) | different species of millet, hemp seeds (*Cannabis sativa*), oat flakes (*Avena sp*) |
|  | Organic soybeans-quarters (Biohof Lex) | soya bean (*Glycine max*) |
|  | Organic oilseeds (OIL + more, Straßberg, Germany) | rapeseeds (*Brassica napus*) |
| Netherlands | Tortelduivenvoer (Wierikx Diervoeders, Roosendaal, Netherlands) | maize (*Zea mays*), wheat (*Tricticum sp*), milo (*Sorghum bicolor*), yellow pea (*Lathyrus aphaca*), pea (*Pisum sativum*), dark pea (*Lathyrus niger*), vetch (*Vicia* *sp*), safflower seeds (*Carthamus tinctorius*) |
|  | Hadivo Tortelduivenvoer (Hadivo Diervoeders B.V., Bourtange, Netherlands) | milo (*Sorghum bicolor*), wheat (*Tricticum sp*), proso millet (*Panicum miliaceum*), buckwheat (*Fagopyrum esculentum*), safflower seeds (*Carthamus tinctorius*), peeled oats (*Avena sp*), mung beans (*Vigna radiate*) |

**Table S4**. Best blast results for each of the 118 detected valid MOTUs using the UniPlant primer pair (Plant) and mICOIintF/dgHCO-2198 (Metazoa) corresponding accession number, the identity with the blast reference sequence, the sequence length and the bitscore. If determination to species level was not clearly determinable, MOTUs were assigned to the lowest shared taxonomic level. Order of the plant MOTUs equates to their order in Table 1

| **MOTU** | **Category** | **Level of determination** | **Accession number** | **Pident (identity) [%]** | **Sequence length [bp]** | **E-value** | **Bitscore** | **Ssciname** |
| --- | --- | --- | --- | --- | --- | --- | --- | --- |
| **Plant** |  |  |  |  |  |  |  |  |
| *Heracleum* sp | natural | genus | EU594907 | 99.663 | 297 | 1.15e-150 | 544 | *Heracleum sphondylium* |
|  |  |  | EU594898 | 99.663 | 297 | 1.15e-150 | 544 | *Heracleum mantegazzianum* |
|  |  |  | EU185658 | 99.663 | 297 | 1.15e-150 | 544 | *Heracleum pubescens* |
| *Hedera* sp | natural | genus | MG218695 | 99.668 | 301 | 2.48e-152 | 549 | *Hedera helix* |
|  |  |  | AJ131236 | 99.666 | 299 | 3.21e-151 | 545 | *Hedera maroccana* |
|  |  |  | AJ131227 | 99.666 | 299 | 3.21e-151 | 545 | *Hedera rhombea* |
| *Achillea* sp | natural | genus | AY603200 | 99.275 | 276 | 2.31e-137 | 499 | *Achillea roseoalba* |
|  |  |  | AY603201 | 98.913 | 276 | 2.99e-136 | 496 | *Achillea aspleniifolia* |
|  |  |  | MG219983 | 98.913 | 276 | 1.08e-135 | 494 | *Achillea millefolium var. lanulosa* |
| *Achillea millefolium* | natural | species | MG219716 | 99.639 | 277 | 1.38e-139 | 507 | *Achillea millefolium* |
| *Bellis perennis* | natural | species | MG219143 | 100.0 | 287 | 8.54e-147 | 531 | *Bellis perennis* |
| *Carthamus tinctorius* | fed | species | MG218972 | 99.317 | 293 | 8.74e-147 | 531 | *Carthamus tinctorius* |
| *Crepis capillaris* | natural | species | MG218876 | 99.672 | 305 | 4.20e-155 | 558 | *Crepis capillaris* |
| *Dittrichia graveolens* | natural | species | MG217717 | 100.0 | 287 | 8.74e-147 | 531 | *Dittrichia graveolens* |
| *Guizotia abyssinica* | fed | species | MG218230 | 99.324 | 296 | 1.91e-148 | 536 | *Guizotia abyssinica* |
| *Helianthus annuus* | fed | species | KY989518 | 99.0 | 300 | 5.37e-149 | 538 | *Helianthus annuus* |
| *Hypochaeris radicata* | natural | species | MF405679 | 99.293 | 283 | 3.04e-141 | 512 | *Hypochaeris radicata* |
| *Lactuca* sp | NA | genus | MK087924 | 98.997 | 299 | 1.93e-148 | 536 | *Lactuca virosa* |
|  |  |  | MG219370 | 98.997 | 299 | 1.93e-148 | 536 | *Lactuca serriola* |
|  |  |  | KY952677 | 98.997 | 299 | 1.93e-148 | 536 | *Lactuca sativa* |
| *Scorzoneroides autumnalis* | natural | species | MG219552 | 99.667 | 300 | 2.48e-152 | 549 | *Scorzoneroides autumnalis* |
| *Senecio inaequidens* | natural | species | JN789801 | 99.662 | 296 | 4.09e-150 | 542 | *Senecio inaequidens* |
| *Sonchus* sp | natural | genus | MH364397 | 99.667 | 300 | 2.48e-152 | 549 | *Sonchus oleraceus* |
|  |  |  | MG256320 | 99.333 | 300 | 1.15e-150 | 544 | *Sonchus arvensis* |
|  |  |  | KY700494 | 99.333 | 300 | 1.15e-150 | 544 | *Sonchus asper* |
| *Artemisia vulgaris* | natural | species | MG218500 | 99.661 | 295 | 1.47e-149 | 540 | *Artemisia vulgaris* |
| *Cichorium* sp | natural | genus | AJ633455 | 99.663 | 297 | 4.10e-150 | 542 | *Cichorium spinosum* |
|  |  |  | AJ746402 | 99.663 | 297 | 4.10e-150 | 542 | *Cichorium intybus* |
|  |  |  | KF241285 | 98.997 | 299 | 6.86e-148 | 534 | *Cichorium glandulosum* |
| *Cirsum* sp | natural | genus | MG218993 | 99.329 | 298 | 1.49e-149 | 540 | *Cirsium vulgare* |
|  |  |  | MG218541 | 99.329 | 298 | 1.49e-149 | 540 | *Cirsium muticum* |
| **MOTU** | **Category** | **Level of determination** | **Accession number** | **Pident (identity) [%]** | **Sequence length [bp]** | **E-value** | **Bitscore** | **Ssciname** |
| *Taraxacum* sp | natural | genus | MK087969 | 99.668 | 301 | 6.92e-153 | 551 | *Taraxacum officinale* |
|  |  |  | MH117786 | 99.668 | 301 | 6.92e-153 | 551 | *Taraxacum dasypodum* |
|  |  |  | MG218717 | 99.668 | 301 | 6.92e-153 | 551 | *Taraxacum latilobum* |
| *Tripleurospermum* sp | natural | genus | MG219864 | 99.275 | 276 | 2.31e-137 | 499 | *Tripleurospermum maritimum* |
|  |  |  | MG740679 | 98.913 | 276 | 2.99e-136 | 496 | *Tripleurospermum kotschyi* |
|  |  |  | MG740700 | 98.913 | 276 | 1.08e-135 | 494 | *Tripleurospermum ziganaense* |
|  |  |  | MG740699 | 98.913 | 276 | 1.08e-135 | 494 | *Tripleurospermum subnivale* |
| *Echium vulgare* | natural | species | KX012025 | 99.663 | 297 | 1.14e-150 | 544 | *Echium vulgare* |
| *Raphanus* sp | brassica | genus | MK424339 | 100.0 | 264 | 4.74e-134 | 488 | *Raphanus sativus* |
|  |  |  | KX709354 | 100.0 | 264 | 4.74e-134 | 488 | *Raphanus raphanistrum* |
| *Sinapis alba* | brassica | species | MG923992 | 99.620 | 263 | 7.89e-132 | 481 | *Sinapis alba* |
| *Brassica* sp | brassica | genus | LR031876 | 99.618 | 262 | 2.83e-131 | 479 | *Brassica oleracea* |
|  |  |  | KX709377 | 99.618 | 262 | 2.83e-131 | 479 | *Brassica carinata* |
| *Brassica juncea* | brassica | species | DQ340633 | 100.0 | 260 | 7.86e-132 | 481 | *Brassica juncea* |
| *Brassica napus* | brassica | species | DQ003664 | 98.473 | 262 | 2.85e-126 | 462 | *Brassica napus* |
| *Brassica oleracea* | brassica | species | LR031874 | 99.627 | 268 | 1.34e-134 | 490 | *Brassica oleracea* |
| *Brassica rapa* | brassica | species | LR031592 | 100.0 | 262 | 6.08e-133 | 484 | *Brassica rapa* |
| *Cardamine hirsuta* | brassica | species | MG886677 | 99.240 | 263 | 3.67e-130 | 475 | *Cardamine hirsuta* |
| *Chenopodium* sp | natural | genus | KU359315 | 99.667 | 300 | 2.48e-152 | 549 | *Chenopodium acerifolium* |
|  |  |  | HM005834 | 99.667 | 300 | 2.48e-152 | 549 | *Chenopodium album* |
| *Cerastium* sp | natural | genus | MH711225 | 99.659 | 293 | 1.88e-148 | 536 | *Cerastium furcatum* |
|  |  |  | MK044717 | 99.659 | 293 | 1.88e-148 | 536 | *Cerastium glomeratum* |
|  |  |  | GU444015 | 99.659 | 293 | 1.88e-148 | 536 | *Cerastium fontanum* |
| *Sagina apetala* | natural | species | MG237249 | 99.658 | 292 | 6.76e-148 | 534 | *Sagina apetala* |
| *Silene* sp | natural | genus | MG237124 | 98.635 | 293 | 1.89e-143 | 520 | *Silene latifolia* |
|  |  |  | MG236292 | 98.635 | 293 | 1.89e-143 | 520 | *Silene noctiflora* |
| *Silene latifolia* | natural | species | DQ005994 | 99.659 | 293 | 1.88e-148 | 536 | *Silene latifolia* |
| *Silene vulgaris* | natural | species | MH333099 | 98.986 | 296 | 8.84e-147 | 531 | *Silene vulgaris* |
| *Stellaria media* | natural | species | MN180184 | 99.658 | 292 | 6.76e-148 | 534 | *Stellaria media* |
| *Cucumis* sp | cultivated | genus | LC435064 | 99.681 | 313 | 1.54e-159 | 573 | *Cucumis melo* |
|  |  |  | AM981116 | 99.681 | 313 | 1.54e-159 | 573 | *Cucumis pubescens* |
| *Cucurbita* sp | cultivated | genus | AM981168 | 98.773 | 326 | 3.46e-161 | 579 | *Cucurbita pepo* |
|  |  |  | FJ915112 | 98.471 | 327 | 4.47e-160 | 575 | *Cucurbita moschata* |
| *Cucurbita pepo* | cultivated | species | MG235650 | 98.765 | 324 | 1.24e-160 | 577 | *Cucurbita pepo* |
| *Sambucus nigra* | tree | species | MG219797 | 99.338 | 302 | 3.23e-151 | 545 | *Sambucus nigra* |
| *Impatiens* sp | natural | genus | MK161039 | 98.496 | 266 | 1.75e-128 | 470 | *Impatiens balfourii* |
|  |  |  | MK161037 | 98.134 | 268 | 6.28e-128 | 468 | *Impatiens parviflora* |
| **MOTU** | **Category** | **Level of determination** | **Accession number** | **Pident (identity) [%]** | **Sequence length [bp]** | **E-value** | **Bitscore** | **Ssciname** |
| *Impatiens parviflora* | natural | species | MK161037 | 99.627 | 269 | 1.34e-134 | 490 | *Impatiens parviflora* |
| *Glycine max* | cultivated | species | MK087915 | 99.303 | 287 | 1.86e-143 | 520 | *Glycine max* |
| *Lotus* sp | natural | genus | HM542536 | 99.655 | 290 | 8.64e-147 | 531 | *Lotus corniculatus* |
|  |  |  | KT250848 | 99.654 | 289 | 3.11e-146 | 529 | *Lotus alpinus* |
|  |  |  | DQ311979 | 99.310 | 290 | 4.02e-145 | 525 | *Lotus tenuis* |
|  |  |  | DQ311974 | 99.310 | 290 | 4.02e-145 | 525 | *Lotus glinoides* |
| *Pisum sativum* | cultivated | species | KU678433 | 99.655 | 290 | 8.64e-147 | 531 | *Pisum sativum* |
| *Robinia* sp | tree | genus | MG236440 | 99.662 | 296 | 4.09e-150 | 542 | *Robinia viscosa* |
|  |  |  | MH711087 | 99.324 | 296 | 1.90e-148 | 536 | *Robinia pseudoacacia* |
| *Trifolium pratense* | natural | species | MK087971 | 99.313 | 291 | 1.12e-145 | 527 | *Trifolium pratense* |
| *Trifolium repens* | natural | species | KR914604 | 99.664 | 298 | 3.18e-151 | 545 | *Trifolium repens* |
| *Vicia* sp | NA | genus | JX506192 | 99.648 | 284 | 1.83e-143 | 520 | *Vicia bungei* |
|  |  |  | MH711085 | 99.298 | 285 | 2.37e-142 | 516 | *Vicia sepium* |
|  |  |  | MG234876 | 99.298 | 285 | 2.37e-142 | 516 | *Vicia lathyroides* |
|  |  |  | KR029993 | 99.298 | 285 | 2.37e-142 | 516 | *Vicia multicaulis* |
| *Vicia hirsuta* | cultivated | species | MG235367 | 99.653 | 288 | 1.11e-145 | 527 | *Vicia hirsuta* |
| *Vicia lathyroides* | natural | species | KJ787168 | 98.947 | 285 | 1.10e-140 | 510 | *Vicia lathyroides* |
| *Vicia sativa* | cultivated | species | KX167836 | 99.649 | 285 | 5.10e-144 | 521 | *Vicia sativa* |
| *Vicia sepium* | cultivated | species | LS973943 | 99.649 | 285 | 5.10e-144 | 521 | *Vicia sepium* |
| *Vicia tetrasperma* | natural | species | KX167838 | 99.653 | 288 | 1.11e-145 | 527 | *Vicia tetrasperma* |
| *Betula* sp | tree | genus | MH711249 | 98.658 | 298 | 3.20e-146 | 529 | *Betula utilis* |
|  |  |  | MH703177 | 98.658 | 298 | 3.20e-146 | 529 | *Betula chinensis* |
|  |  |  | MK453282 | 98.658 | 298 | 3.20e-146 | 529 | *Betula costata* |
|  |  |  | AY761118 | 98.658 | 298 | 3.20e-146 | 529 | *Betula maximowicziana* |
|  |  |  | AY761101 | 98.658 | 298 | 3.20e-146 | 529 | *Betula alnoides* |
| *Carpinus* sp | tree | genus | KX167948 | 99.666 | 299 | 8.88e-152 | 547 | *Carpinus betulus* |
|  |  |  | KX946974 | 98.0 | 300 | 1.94e-143 | 520 | *Carpinus langaoensis* |
| *Fagus* sp | tree | genus | AY232981 | 99.007 | 302 | 4.19e-150 | 542 | *Fagus sylvatica* |
|  |  |  | MH711699 | 98.344 | 302 | 2.52e-147 | 532 | *Fagus engleriana* |
|  |  |  | LT984625 | 98.344 | 302 | 9.08e-147 | 531 | *Fagus lucida* |
| *Juglans regia* | tree | species | MH712684 | 99.660 | 294 | 5.24e-149 | 538 | *Juglans regia* |
| *Galium* sp | natural | genus | AF419185 | 98.730 | 315 | 4.38e-155 | 558 | *Galium aparine* |
|  |  |  | MG905992 | 98.714 | 311 | 2.08e-153 | 553 | *Galium pamiroalaicum* |
| *Hippuris* sp | natural | genus | MG220001 | 99.293 | 283 | 3.06e-141 | 512 | *Hippuris x lanceolata* |
|  |  |  | MG219603 | 99.293 | 283 | 3.06e-141 | 512 | *Hippuris tetraphylla* |
| *Plantago lanceolata* | natural | species | MG256295 | 99.286 | 280 | 1.40e-139 | 507 | *Plantago lanceolata* |
| *Veronica chamaedrys* | natural | species | MG217702 | 99.653 | 288 | 1.11e-145 | 527 | *Veronica chamaedrys* |
| **MOTU** | **Category** | **Level of determination** | **Accession number** | **Pident (identity) [%]** | **Sequence length [bp]** | **E-value** | **Bitscore** | **Ssciname** |
| *Lilium* sp | NA | genus | FJ979907 | 99.351 | 308 | 1.18e-155 | 560 | *Lilium martagon* |
|  |  |  | KC020198 | 99.351 | 308 | 4.24e-155 | 558 | *Lilium distichum* |
|  |  |  | FJ979904 | 99.026 | 308 | 1.53e-154 | 556 | *Lilium cattaniae* |
| *Euphorbia helioscopia* | natural | species | MH107040 | 99.656 | 291 | 2.41e-147 | 532 | *Euphorbia helioscopia* |
| *Mercurialis annua* | natural | species | MK602636 | 99.656 | 291 | 2.41e-147 | 532 | *Mercurialis annua* |
| *Linum* sp | cultivated | genus | MH592580 | 99.652 | 287 | 3.97e-145 | 525 | *Linum usitatissimum* |
|  |  |  | MG236207 | 99.652 | 287 | 3.97e-145 | 525 | *Linum bienne* |
| *Tilia* sp | tree | genus | MG237456 | 99.678 | 311 | 2.00e-158 | 569 | *Tilia americana* |
|  |  |  | AF174639 | 99.035 | 311 | 3.34e-156 | 562 | *Tilia caroliniana* |
|  |  |  | LS973936 | 99.035 | 311 | 4.32e-155 | 558 | *Tilia cordata* |
| *Tilia platyphyllos* | tree | species | LS999888 | 99.361 | 313 | 7.18e-158 | 568 | *Tilia platyphyllos* |
| *Lythrum salicaria* | natural | species | MH711465 | 99.658 | 292 | 6.73e-148 | 534 | *Lythrum salicaria* |
| *Epilobium* sp | natural | genus | MG237828 | 99.652 | 287 | 3.97e-145 | 525 | *Epilobium parviflorum* |
|  |  |  | MG235668 | 99.652 | 287 | 3.97e-145 | 525 | *Epilobium coloratum* |
|  |  |  | MG235125 | 99.652 | 287 | 3.97e-145 | 525 | *Epilobium ciliatum* |
|  |  |  | KX166817 | 99.652 | 287 | 3.97e-145 | 525 | *Epilobium obscurum* |
| *Oenothera* sp | natural | genus | MH712700 | 99.650 | 286 | 1.42e-144 | 523 | *Oenothera biennis* |
|  |  |  | LC365303 | 99.650 | 286 | 1.42e-144 | 523 | *Oenothera stricta* |
|  |  |  | MG237526 | 99.650 | 286 | 1.42e-144 | 523 | *Oenothera clelandii* |
|  |  |  | MG237158 | 99.650 | 286 | 1.42e-144 | 523 | *Oenothera villosa* |
|  |  |  | MG236992 | 99.650 | 286 | 1.42e-144 | 523 | *Oenothera parviflora* |
| *Picea* sp | tree | genus | MG216912 | 99.676 | 309 | 2.54e-157 | 566 | *Picea mariana* |
|  |  |  | MG216899 | 99.676 | 309 | 2.54e-157 | 566 | *Picea engelmannii* |
|  |  |  | MG216855 | 99.676 | 309 | 2.54e-157 | 566 | *Picea sitchensis* |
|  |  |  | MF349026 | 99.676 | 309 | 2.54e-157 | 566 | *Picea glauca* |
| *Pinus* sp | tree | genus | MG216921 | 100.0 | 315 | 2.58e-162 | 582 | *Pinus monticola* |
|  |  |  | MG216819 | 100.0 | 315 | 2.58e-162 | 582 | *Pinus flexilis* |
|  |  |  | AY430077 | 100.0 | 315 | 2.58e-162 | 582 | *Pinus sibirica* |
|  |  |  | AF036981 | 100.0 | 315 | 2.58e-162 | 582 | *Pinus ayacahuite* |
| *Pinus sylvestris* | tree | species | MG216892 | 99.684 | 316 | 3.35e-161 | 579 | *Pinus sylvestris* |
| *Carex* sp | natural | genus | MG216748 | 98.154 | 325 | 2.08e-158 | 569 | *Carex assiniboinensis* |
|  |  |  | MG215766 | 98.154 | 325 | 7.49e-158 | 568 | *Carex lurida* |
| Poaceae | NA | family | KF482108 | 99.656 | 291 | 2.41e-147 | 532 | *Triticum aestivum* |
|  |  |  | HQ600533 | 99.656 | 291 | 2.41e-147 | 532 | *Secale cereale* |
| *Agrostis* sp | natural | genus | KU883481 | 100.0 | 289 | 6.65e-148 | 534 | *Agrostis castellana* |
|  |  |  | FJ042825 | 100.0 | 289 | 6.65e-148 | 534 | *Agrostis capillaris* |
|  |  |  | KX166225 | 99.654 | 289 | 8.61e-147 | 531 | *Agrostis gigantea* |
| **MOTU** | **Category** | **Level of determination** | **Accession number** | **Pident (identity) [%]** | **Sequence length [bp]** | **E-value** | **Bitscore** | **Ssciname** |
| *Alopecurus myosuroides* | natural | species | MG215855 | 99.310 | 290 | 4.02e-145 | 525 | *Alopecurus myosuroides* |
| *Alopecurus pratensis* | natural | species | MG216008 | 99.310 | 290 | 4.02e-145 | 525 | *Alopecurus pratensis* |
| *Arrhenatherum* sp | natural | genus | KP295984 | 99.654 | 289 | 3.10e-146 | 529 | *Arrhenatherum elatius* |
|  |  |  | AJ632184 | 99.654 | 289 | 3.10e-146 | 529 | *Arrhenatherum album* |
| *Arrhenatherum elatius* | natural | species | KU883487 | 99.306 | 288 | 5.18e-144 | 521 | *Arrhenatherum elatius* |
| *Avena* sp | NA | genus | AY522436 | 99.653 | 288 | 1.11e-145 | 527 | *Avena longiglumis* |
|  |  |  | MF029731 | 99.649 | 285 | 5.16e-144 | 521 | *Avena fatua* |
|  |  |  | MG216317 | 99.649 | 285 | 5.16e-144 | 521 | *Avena sterilis* |
| *Dactylis glomerata* | natural | species | MG216301 | 99.308 | 289 | 1.44e-144 | 523 | *Dactylis glomerata* |
| *Elymus* sp | natural | genus | FJ793078 | 99.655 | 290 | 8.67e-147 | 531 | *Elymus repens* |
|  |  |  | MK721822 | 99.310 | 290 | 4.04e-145 | 525 | *Elymus lolioides* |
| *Festuca* sp | natural | genus | KY999968 | 99.654 | 289 | 3.10e-146 | 529 | *Festuca kirelowii* |
|  |  |  | KX166185 | 99.654 | 289 | 3.10e-146 | 529 | *Festuca rubra* |
|  |  |  | MK483090 | 99.308 | 289 | 4.00e-145 | 525 | *Festuca rubra x Festuca arctica* |
| *Holcus* sp | natural | genus | KU883508 | 100.0 | 289 | 6.68e-148 | 534 | *Holcus lanatus* |
|  |  |  | KX166166 | 100.0 | 289 | 6.68e-148 | 534 | *Holcus mollis* |
| *Hordeum vulgare* | cultivated | species | LR722623 | 100.0 | 292 | 1.45e-149 | 540 | *Hordeum vulgare* |
| *Lolium* sp | natural | genus | KP296068 | 99.306 | 288 | 5.18e-144 | 521 | *Lolium perenne* |
|  |  |  | MG216281 | 99.306 | 288 | 5.18e-144 | 521 | *Lolium rigidum* |
|  |  |  | KP205444 | 99.306 | 288 | 5.18e-144 | 521 | *Lolium multiflorum* |
| *Lolium perenne* | natural | species | KX167358 | 100.0 | 288 | 2.39e-147 | 532 | *Lolium perenne* |
| *Molinia caerulea* | natural | species | MG215651 | 100.0 | 325 | 7.38e-168 | 601 | *Molinia caerulea* |
| *Panicum miliaceum* | fed | species | JX576540 | 100.0 | 292 | 1.45e-149 | 540 | *Panicum miliaceum* |
| *Phalaris* sp | natural | genus | KX873131 | 100.0 | 290 | 1.86e-148 | 536 | *Phalaris minor* |
|  |  |  | KX873133 | 100.0 | 289 | 6.68e-148 | 534 | *Phalaris paradoxa* |
| *Poa* sp | natural | genus | KP296096 | 99.649 | 285 | 5.10e-144 | 521 | *Poa compressa* |
|  |  |  | GQ324521 | 99.298 | 285 | 2.37e-142 | 516 | *Poa lettermanii* |
|  |  |  | EU792398 | 99.298 | 285 | 2.37e-142 | 516 | *Poa pseudoabbreviata* |
| *Poa trivialis* | natural | species | KP296102 | 99.652 | 287 | 3.97e-145 | 525 | *Poa trivialis* |
| *Secale cereale* | cultivated | species | KP296112 | 100.0 | 290 | 1.86e-148 | 536 | *Secale cereale* |
| *Setaria* sp | fed | genus | KF012848 | 99.315 | 292 | 3.13e-146 | 529 | *Setaria italica* |
|  |  |  | MH711286 | 98.973 | 292 | 1.46e-144 | 523 | *Setaria viridis* |
|  |  |  | MG216638 | 98.973 | 292 | 1.46e-144 | 523 | *Setaria faberi* |
| *Trisetum flavescens* | cultivated | species | MG216670 | 99.658 | 292 | 6.73e-148 | 534 | *Trisetum flavescens* |
| *Triticum* sp | cultivated | genus | KC589715 | 99.315 | 292 | 3.13e-146 | 529 | *Triticum aestivum* |
|  |  |  | MH618755 | 98.973 | 292 | 1.46e-144 | 523 | *Triticum turgidum* |
|  |  |  | MH618737 | 98.973 | 292 | 1.46e-144 | 523 | *Triticum dicoccon* |
| **MOTU** | **Category** | **Level of determination** | **Accession number** | **Pident (identity) [%]** | **Sequence length [bp]** | **E-value** | **Bitscore** | **Ssciname** |
| *Triticum aestivum* | cultivated | species | FM998923 | 99.658 | 292 | 6.73e-148 | 534 | *Triticum aestivum* |
| *Triticum dicoccon* | cultivated | species | MH618738 | 98.282 | 291 | 1.13e-140 | 510 | *Triticum dicoccon* |
| *Triticum spelta* | cultivated | species | KJ131570 | 99.194 | 248 | 9.02e-122 | 448 | *Triticum spelta* |
| *Zea mays* | cultivated | species | MG215771 | 99.065 | 321 | 3.46e-161 | 579 | *Zea mays* |
| *Ranunculus* sp | natural | genus | MG237407 | 100.0 | 278 | 8.33e-142 | 514 | *Ranunculus macounii* |
|  |  |  | KX166649 | 100.0 | 278 | 8.33e-142 | 514 | *Ranunculus sardous* |
| *Cannabis sativa* | fed | species | MF972942 | 99.658 | 292 | 6.73e-148 | 534 | *Cannabis sativa* |
| *Hippophae rhamnoides* | NA | species | KY411029 | 99.658 | 292 | 6.73e-148 | 534 | *Hippophae rhamnoides* |
| *Amelanchier* sp | tree | genus | KY193975 | 99.653 | 288 | 1.11e-145 | 527 | *Amelanchier sp. 'rubra'* |
|  |  |  | MG236109 | 99.306 | 288 | 5.16e-144 | 521 | *Amelanchier alnifolia* |
| *Potentilla* sp | natural | genus | MG235863 | 99.286 | 280 | 1.40e-139 | 507 | *Potentilla reptans* |
|  |  |  | KX167692 | 99.286 | 280 | 1.40e-139 | 507 | *Potentilla anglica* |
|  |  |  | KX167462 | 99.286 | 280 | 1.40e-139 | 507 | *Potentilla recta* |
| *Prunus* sp | tree | genus | AF318731 | 99.644 | 281 | 2.34e-142 | 516 | *Prunus pseudocerasus* |
|  |  |  | KY749311 | 99.644 | 281 | 8.43e-142 | 514 | *Prunus avium* |
| *Prunus avium* | tree | species | KY749326 | 98.936 | 282 | 5.07e-139 | 505 | *Prunus avium* |
| *Rosa* sp | natural | genus | LS973926 | 99.296 | 284 | 8.50e-142 | 514 | *Rosa canina* |
|  |  |  | KX167433 | 99.296 | 284 | 8.50e-142 | 514 | *Rosa obtusifolia* |
|  |  |  | KX166516 | 99.296 | 284 | 8.50e-142 | 514 | *Rosa micrantha* |
|  |  |  | KX165942 | 99.296 | 284 | 8.50e-142 | 514 | *Rosa arvensis* |
|  |  |  | KX165933 | 99.296 | 284 | 8.50e-142 | 514 | *Rosa tomentosa* |
| *Rubus* sp | natural | genus | KX166132 | 99.645 | 282 | 2.34e-142 | 516 | *Rubus plicatus* |
|  |  |  | KM037649 | 99.645 | 282 | 2.34e-142 | 516 | *Rubus wimmerianus* |
|  |  |  | KM037600 | 99.645 | 282 | 2.34e-142 | 516 | *Rubus sanctus* |
|  |  |  | KM037591 | 99.645 | 282 | 2.34e-142 | 516 | *Rubus tabanimontanus* |
|  |  |  | KM037489 | 99.645 | 282 | 2.34e-142 | 516 | *Rubus praecox* |
| *Urtica dioica* | natural | species | MK087974 | 99.685 | 317 | 9.35e-162 | 580 | *Urtica dioica* |
| *Acer* sp | tree | genus | AY605409 | 99.671 | 304 | 1.50e-154 | 556 | *Acer negundo* |
|  |  |  | AM238326 | 99.342 | 304 | 6.99e-153 | 551 | *Acer saccharum* |
| *Acer platanoides* | tree | species | LK022679 | 99.676 | 309 | 2.55e-157 | 566 | *Acer platanoides* |
| *Acer pseudoplatanus* | tree | species | AM238276 | 99.681 | 313 | 1.54e-159 | 573 | *Acer pseudoplatanus* |
| *Sedum* sp | natural | genus | MG236076 | 99.308 | 289 | 5.16e-144 | 521 | *Sedum sexangulare* |
|  |  |  | MG235623 | 98.962 | 289 | 6.67e-143 | 518 | *Sedum acre* |
| *Convolvulus arvensis* | natural | species | MH189723 | 99.664 | 298 | 3.18e-151 | 545 | *Convolvulus arvensis* |
| *Solanum lycopersicum* | cultivated | species | CP023767 | 100.0 | 280 | 6.49e-143 | 518 | *Solanum lycopersicum* |
|  |  |  |  |  |  |  |  |  |
|  |  |  |  |  |  |  |  |  |
|  |  |  |  |  |  |  |  |  |
| **MOTU** | **Category** | **Level of determination** | **Accession number** | **Pident (identity) [%]** | **Sequence length [bp]** | **E-value** | **Bitscore** | **Ssciname** |
| **Metazoa** |  |  |  |  |  |  |  |  |
| *Agriopis leucophaearia* |  | species | JN269325 | 99.042 | 313 | 3.34e-156 | 562 | *Agriopis leucophaearia* |
| *Anthomyia* sp |  | genus | MG116630 | 100.0 | 313 | 3.32e-161 | 579 | *Anthomyia sp.* BIOUG01544-C05 |
| *Dolichogenidea sp* |  | genus | HM397208 | 100.0 | 313 | 3.32e-161 | 579 | *Dolichogenidea sp.* BOLD:AAA5831 |
| *Eudemis profundana* |  | species | KX049115 | 99.042 | 313 | 3.34e-156 | 562 | *Eudemis profundana* |
| Hemiptera |  | order | MF928832 | 99.361 | 313 | 7.18e-158 | 568 | Hemiptera sp. BIOUG03768-E03 |
| *Lumbricus terrestris* |  | species | MG421482 | 100.0 | 313 | 3.32e-161 | 579 | *Lumbricus terrestris* |
| *Taeniothrips inconsequens* |  | species | KR144423 | 100.0 | 310 | 1.53e-159 | 573 | *Taeniothrips inconsequens* |

**Table S5**. Common Woodpigeon *Columba palumbus* diet composition: Compilation of our results and results from previous studies. Given are the plant families and animal prey taxa found in the diet of Woodpigeons based on different methods. The plant or animal item is marked with “x” if it was found in the respective study

|  | Present study | Kaouachi  et al. 2021 | Dunn  et al. 2018 | Gutiérrez-Galán  et al. 2017 | Merabet et al. 2014 | Ó hUallachain & Dunne 2013 | Herland 2007 | Jimenez  et al. 1994 | Folk 1984 | Lebeurier 1963 | Murton  et al. 1962 | Murton  et al. 1964 | Collinge  1913 | Bettmann 1966 | Niethammer & Przygodda 1954^a^ |
| --- | --- | --- | --- | --- | --- | --- | --- | --- | --- | --- | --- | --- | --- | --- | --- |
| **Method** | molecular  (faeces) | visual (crop) + observation | molecular  (faeces) | visual (crop) | visual (stomach) | visual (crop) | visual (crop) | visual (digestive tract) | visual (crop + stomach) | visual (crop + stomach) | visual (crop) | visual (crop) | visual (digestive tract) | visual (crop) | visual (crop) |
| **Sampling country** | DE | DZ | UK | ES | DZ | IE | NO | ES | CZ | FR | UK | UK | UK | DE | DE |
| **Sampling years** | 2018-20 | 2015-17 | 2011-14 | 2011/12 | 2006/7 | 2000-02 | 2005 | 1987-89 | 1968-72 | 1935-61 | 1957-60 | 1958-60 | NA | 1963-65 | 1953 |
| **Sample size** | n = 49 | n = 86 | n = 5 | n = 206 | N = 36 | n = 299 | n = 72 | n = 48 | n = 112 | n = 67 | n = 82^b^ | n = 614 | n = 388 | n = 360 | n = 65 |
| Plant family |  |  |  |  |  |  |  |  |  |  |  |  |  |  |  |
| Adoxaceae | x |  |  |  |  | x |  |  |  |  | x | x |  |  |  |
| Amaranthaceae | x |  | x |  |  |  |  |  |  |  |  | x |  |  |  |
| Amaryllidaceae |  |  |  |  |  |  |  | x |  |  |  |  |  |  |  |
| Anacardiaceae |  | x |  | x | x |  |  |  |  |  |  |  |  |  |  |
| Apiaceae |  |  | x |  |  |  |  |  | x |  |  |  |  |  |  |
| Aquifoliaceae |  |  |  |  |  |  |  |  |  | x |  |  | x |  |  |
| Araliaceae | x | x |  |  |  | x |  |  |  | x | x | x |  |  |  |
| Asparagaceae |  |  |  |  |  |  |  | x |  |  |  |  |  |  |  |
| Asteraceae | x |  | x | x | x | x | x | x | x | x | x |  |  | x |  |
| Betulaceae | x |  |  |  |  |  |  |  |  | x |  | x |  |  |  |
| Boraginaceae |  |  | x |  |  |  |  |  |  |  |  |  |  |  |  |
| Brassicaceae | x |  | x | x | x | x | x |  | x | x | x | x | x | x | x |
| Calymperaceae |  |  |  |  |  | x |  |  |  |  |  |  |  |  |  |
| Cannabaceae | x | x |  |  |  |  |  |  |  |  |  |  |  |  |  |
| Caryophyllaceae | x |  | x |  |  | x | x |  | x | x | x | x | x | x |  |
| Celastraceae |  |  |  |  |  |  |  |  |  | x |  |  |  |  |  |
| Convolvulaceae | x |  | x | x |  |  |  | x |  |  |  |  |  |  |  |
| Crassulaceae | x |  |  |  |  |  |  |  |  |  |  |  |  |  |  |
| Cucurbitaceae | x |  | x |  |  |  |  |  |  |  |  |  |  |  |  |
| Cyperaceae | x |  |  |  |  | x | x |  |  |  |  |  |  |  |  |
| Euphorbiaceae |  |  |  |  |  |  |  | x |  |  |  |  |  |  |  |
| Fabaceae | x | x |  | x | x | x | x | x | x | x | x | x | x | x | x |
| Fagaceae | x | x |  |  | x | x |  |  | x | x | x | x | x | x |  |
| Geraniaceae |  |  | x |  |  |  |  |  |  | x |  |  |  |  |  |
| Juglandaceae | x |  |  |  |  |  |  |  |  |  |  |  |  |  |  |
| Lamiaceae |  |  |  |  |  |  |  |  | x | x | x |  |  | x | x |
| Lembophyllaceae |  |  |  |  |  |  |  |  |  | x |  |  |  |  |  |
| Liliaceae | x |  |  |  |  |  |  |  |  |  |  |  |  |  |  |
|  |  |  |  |  |  |  |  |  |  |  |  |  |  |  |  |
|  | Present study | Kaouachi  et al. 2021 | Dunn  et al. 2018 | Gutiérrez-Galán  et al. 2017 | Merabet et al. 2014 | Ó hUallachain & Dunne 2013 | Herland 2007 | Jimenez  et al. 1994 | Folk 1984 | Lebeurier 1963 | Murton  et al. 1962 | Murton  et al. 1964 | Collinge  1913 | Bettmann 1966 | Niethammer & Przygodda 1954 |
| Linaceae |  |  |  |  |  | x |  |  |  |  |  |  |  |  |  |
| Malvaceae | x |  |  |  |  |  |  |  |  |  |  |  |  |  |  |
| Myrtaceae |  |  |  |  | x |  |  |  |  |  |  |  |  |  |  |
| Oleaceae |  | x |  | x | x |  |  | x |  |  | x | x |  |  |  |
| Onagraceae |  |  |  |  |  |  |  |  |  |  |  |  |  | x |  |
| Oxalidaceae |  |  |  |  | x |  |  |  |  |  |  |  |  |  |  |
| Papaveraceae |  |  |  |  |  |  |  |  |  | x |  |  |  |  |  |
| Pinaceae | x | x |  |  |  |  |  |  | x |  |  |  |  | x |  |
| Plantaginaceae | x |  |  |  |  |  |  |  | x | x | x | x |  | x |  |
| Poaceae | x | x | x | x | x | x | x | x | x | x | x | x | x | x | x |
| Polygonaceae |  |  |  |  |  | x |  |  | x | x | x |  | x | x |  |
| Primulaceae |  |  | x |  |  | x |  |  |  | x |  |  |  |  |  |
| Ranunculaceae |  |  |  |  |  | x |  |  |  | x | x | x | x | x | x |
| Resedaceae |  |  | x |  |  |  |  |  |  |  |  |  |  |  |  |
| Rosaceae | x |  | x |  | x | x | x |  | x | x | x | x | x | x | x |
| Rubiaceae | x |  |  | x |  | x |  |  | x |  | x |  |  |  |  |
| Sapindaceae | x |  |  |  |  |  |  |  |  |  |  |  |  |  |  |
| Salicaceae |  |  |  |  |  | x |  |  |  |  |  | x |  |  |  |
| Solanaceae | x |  |  |  | x | x |  |  |  |  |  |  | x | x |  |
| Ulmaceae |  |  |  |  | x |  |  |  |  |  |  |  |  |  |  |
| Urticaceae | x |  |  |  |  | x |  |  |  |  |  |  |  | x | x |
| Violaceae |  |  | x |  |  | x |  |  |  |  | x | x |  | x |  |
| **No. families** | 25 | 8 | 14 | 8 | 12 | 20 | 7 | 8 | 13 | 19 | 16 | 15 | 10 | 16 | 7 |
|  |  |  |  |  |  |  |  |  |  |  |  |  |  |  |  |
| Animal prey |  |  | Not analysed |  |  |  |  |  |  |  |  |  | Not mentioned |  |  |
| Gastropoda |  |  | - | x | x | x | x |  | x | x | x | x | - | x |  |
| Clitellata |  |  | - |  |  | x | x |  | x | x |  |  | - | x |  |

^a^ weed seeds were not specified in detail

^b^ all nestlings

References:

Bettmann H (1966) Untersuchungen der Kropfinhalte von Ringeltauben. Zeitschrift für Jagdwissenschaft 12:97–125. https://doi.org/10.1007/BF02316513

Collinge WE (1913) The food of some British wild birds: a study in economic ornithology. Dulau & Co., London.

Dunn JC, Stockdale JE, Moorhouse-Gann RJ, et al. (2018) The decline of the Turtle Dove: Dietary associations with body condition and competition with other columbids analysed using high‐throughput sequencing. Mol Ecol 27:3386–3407. https://doi.org/10.1111/mec.14766

Folk C (1984) The summer diet of the woodpigeon. Folia Zool 33:41–47.

Gutiérrez-Galán A, González CA, de Mercado JM (2017) Woodpigeon *Columba palumbus* diet compositions in Mediterranean Southern Spain. Ardeola 64:17–30. https://doi.org/10.13157/arla.64.1.2017.ra2

Herland A (2007) Relationship between flocking, diet and morphology in woodpigeons (*Columba palumbus*) during early autumn in South-Eastern Norway. Master thesis, Norwegian University of life sciences (NMBU), Department of ecology and natural resource management.

Jimenez R, Hodar JA, Camacho I (1994) Diet of the woodpigeon (*Columba palumbus*) in the south of Spain during late summer. Fol Zool 43:163–170.

Kaouachi A, Menaa M, Rebbah AC, Maazi MC (2021) Diet of Wood Pigeon (*Columba palumbus*) in Forest Areas of Souk Ahras Region (North-Eastern Algeria): Management Implications. Pakistan J Zool 53:1603-2000. https://dx.doi.org/10.17582/journal.pjz/20190708150749.

Lebeurier E (1963) Régime alimentaire du Pigeon ramier dans les arrondissements de Morlaix et de Châteaulin (Finistère). L’Oiseau et R.F.O. 33:212–234.

Merabet A, Chebouti-Meziou N, Chebouti Y, Bissaad FZ, Doumandji S (2014) Le régime alimentaire du Pigeon ramier *Columba palumbus* aux abords de la plaine de la Mitidja (Nord Algérie). Rev Écol 69:247–257.

Murton RK, Isaacson AJ, Westwood NJ (1962) The food and growth of nestling wood-pigeons in relation to the breeding season. J Zool 141:747–781. https://doi.org/10.1111/j.1469-7998.1963.tb01624.x.

Murton RK, Westwood NJ, Isaacson A (1964) The feeding habits of the woodpigeon *Columba palumbus*, Stock Dove *C. oenas* and Turtle Dove *Streptopelia turtur*. Ibis 106:174–188.

Niethammer G, Przygodda W (1954) Zur Ernährung von Ringel- und Hohltaube. Beobachtungen an einem Schlafplatz bei Bonn. Die Vogelwelt 75:41–55.

Ó hUallachain D, Dunne J (2013) Seasonal variation in the diet and food preference of the Woodpigeon *Columba palumbus* in Ireland. Bird Study 60:417–422. https://doi.org/10.1080/00063657.2013.798259.

**Table S6**. European Turtle Dove *Streptopelia turtur* diet composition: Compilation of our results and results from previous studies. Given are the plant families and animal prey taxa found in the diet of Turtle Doves based on different methods. The plant or animal item is marked with “x” if it was found in the respective study

|  | Present study | Mansouri  et al. 2019^a^ | Dunn  et al. 2018 | Gutiérrez-Galán & Alonso 2016 | Browne  & Aebischer 2003a | Calladine et al. 1997 | Dias  & Fontoura 1996 | Jimenez  et al. 1992 | Glutz von Blotzheim & Bauer 1987^b^ | Glutz von Blotzheim & Bauer 1987^c^ | Bijlsma 1985 | Kiss  et al. 1978^d^ | Murton  et al. 1965^e^ | Murton  et al. 1964 |
| --- | --- | --- | --- | --- | --- | --- | --- | --- | --- | --- | --- | --- | --- | --- |
| **Method** | molecular  (faeces) | visual (digestive tract) | molecular  (faeces) | visual (digestive tract) | visual (faeces) | Visual (faeces) | visual  (crop + gizzard) | visual (digestive tract) | visual (stomach) | visual (digestive tract) | observation | visual (stomach) | visual  (crop + gizzard) | visual  (crop) |
| **Sampling country** | DE + NL | MA | UK | ES | UK | UK | PT | ES | HU | SK | NL | RO | RU | UK |
| **Sampling years** | 2018-20 | 2016/17 | 2011-14 | 2013-15 | 1998-2000 | 1996 | 1991-93 | 1988-90 | NA | NA | 1977-81 | NA | 1937-39 | 1961/62 |
| **Sample size** | n = 19 | n = 68 | n = 54 | n = 222 | n = 71^f^ | n = 18 | n = 110 | n = 64 | n = 36 | n = 44 | NA | n = 22 | n = 107 | n = 46 |
| Plant family |  |  |  |  |  |  |  |  |  |  |  |  |  |  |
| Adoxaceae |  |  | x |  |  |  |  |  |  |  |  |  |  |  |
| Amaranthaceae | x |  | x | x | x |  | x | x | x | x |  | x | x | x |
| Apiaceae | x |  | x |  |  |  |  |  |  |  |  |  |  |  |
| Asteraceae | x |  | x | x |  |  | x | x |  | x |  | x | x | x |
| Betulaceae | x |  |  |  |  |  |  |  |  |  |  |  |  |  |
| Boraginaceae | x |  | x | x |  |  |  |  |  |  |  |  |  |  |
| Brassicaceae | x |  | x | x | x | x | x | x | x | x | x | x |  | x |
| Cannabaceae | x |  | x |  |  |  |  |  |  |  |  | x |  |  |
| Caprifoliaceae |  |  |  |  |  |  | x |  |  |  |  |  |  |  |
| Caryophyllaceae |  |  | x | x | x | x |  |  |  |  | x |  |  | x |
| Cistaceae |  |  |  |  |  |  |  | x |  |  |  |  |  |  |
| Convolvulaceae |  |  | x | x |  |  | x | x |  |  |  | x |  |  |
| Crassulaceae |  |  | x |  |  |  |  |  |  |  |  |  |  |  |
| Cucurbitaceae | x |  | x |  |  |  |  |  |  |  |  | x |  |  |
| Cupressaceae |  |  | x |  |  |  |  |  |  |  |  |  |  |  |
| Cyperaceae | x |  |  |  |  |  |  |  |  |  |  |  |  |  |
| Euphorbiaceae | x |  | x | x |  |  |  | x |  |  |  | x | x | x |
| Fabaceae | x |  | x | x |  |  | x | x | x | x |  | x | x | x |
| Geraniaceae |  |  | x | x |  |  |  |  |  |  |  |  |  |  |
| Hypericaceae |  |  |  |  |  |  | x |  |  |  |  |  |  |  |
| Lamiaceae |  |  |  |  |  |  |  |  | x |  |  | x |  |  |
| Linaceae | x |  | x |  |  | x |  |  |  |  |  |  |  |  |
| Lythraceae | x |  |  |  |  |  |  |  |  |  |  |  |  |  |
| Malvaceae |  |  |  | x |  |  |  |  |  |  |  |  |  |  |
| Onagraceae | x |  | x |  |  |  |  |  |  |  |  |  |  |  |
|  |  |  |  |  |  |  |  |  |  |  |  |  |  |  |
|  |  |  |  |  |  |  |  |  |  |  |  |  |  |  |
|  | Present study | Mansouri  et al. 2019^a^ | Dunn  et al. 2018 | Gutiérrez-Galán & Alonso 2016 | Browne  & Aebischer 2003 | Calladine et al. 1997 | Dias  & Fontoura 1996 | Jimenez  et al. 1992 | Glutz von Blotzheim & Bauer 1987^b^ | Glutz von Blotzheim & Bauer 1987^c^ | Bijlsma 1985 | Kiss  et al. 1978^d^ | Murton  et al. 1965 | Murton  et al. 1964 |
| Papaveraceae |  |  | x |  | x |  | x |  | x |  | x | x | x | x |
| Pinaceae | x |  | x |  |  |  |  |  |  |  | x |  |  |  |
| Plantaginaceae |  |  | x |  |  |  |  |  |  |  |  | x |  | x |
| Poaceae | x | x | x | x | x | x | x | x | x | x | x | x | x | x |
| Polygonaceae |  |  | x | x | x |  | x |  |  | x | x | x | x | x |
| Primulaceae |  |  | x |  |  |  |  |  |  |  |  |  |  | x |
| Ranunculaceae | x |  | x | x |  | x |  |  |  |  |  |  | x | x |
| Resedaceae |  |  |  |  | x |  |  |  |  |  |  | x |  | x |
| Rhamnaceae |  |  | x |  |  |  |  |  |  |  |  |  |  |  |
| Rosaceae | x |  | x |  |  |  |  |  |  |  |  |  | x |  |
| Rubiaceae |  |  | x | x |  |  |  |  |  |  |  |  |  | x |
| Rutaceae |  |  | x |  |  |  |  |  |  |  |  |  |  |  |
| Sapindaceae |  |  | x |  |  |  |  |  |  |  |  |  |  |  |
| Urticaceae | x |  | x |  | x | x |  |  |  |  |  |  |  |  |
| Violaceae |  |  | x |  | x |  |  |  |  |  |  |  |  | x |
| **No. families** | 19 | 1 | 31 | 14 | 9 | 6 | 10 | 8 | 6 | 6 | 6 | 14 | 9 | 15 |
|  |  |  |  |  |  |  |  |  |  |  |  |  |  |  |
| Animal prey |  |  | Not analysed |  | Not mentioned |  |  |  | Not mentioned | Not mentioned |  | Not specified^g^ |  |  |
| Gastropoda |  | x | - | x | - |  | x | x | - | - |  | - | x | x |
| Bivalvia |  |  | - |  | - |  |  |  | - | - |  | - | x |  |

^a^ a moderate proportion of elements (7.32%) remained undefined. Only soft wheat, barley seeds and gastropod shells were identified

^b^ information from Sterbetz I. by letter

^c^ data from Feriancová Z. (1955) Die Nahrung der Türkentaube und der Turteltaube. Biologia 10: 436–449

^d^ complemented with information from Kiss J.B. and Sterbetz I. by letter for 29 Turtle Dove individuals mentioned in Glutz von Blotzheim & Bauer 1987

^e^ data from a Russian paper by Likhachev G.N. (1954) Byulleten ILI. 0-va Isp. Prirodi Otd. Biol. T. 15-25. Percentages of less than 10 were not included

^f^ 18 samples from adult Turtle Doves and 53 samples collected from nests

^g^ unidentified Mollusca in two individuals

References:

Bijlsma RG (1985) De broedbiologie van de Tortelduif *Streptopelia turtur*. Het Vogeljaar 33:225–532.

Browne SJ, Aebischer NJ (2003) Habitat use, foraging ecology and diet of turtle doves *Streptopelia turtur* in Britain. Ibis 145:572–582. https://doi.org/10.1046/j.1474-919X.2003.00185.x.

Calladine JR, Buner F, Aebischer NJ (1997) The summer ecology and habitat use of the Turtle Dove: A pilot study. English Nature.

Dias S, Fontoura AP (1996) A dieta estival da rôla-brava (*Streptopelia turtur*) no sul de Portugal. Revista Forestal 9:227–241.

Dunn JC, Stockdale JE, Moorhouse-Gann RJ, et al. (2018) The decline of the Turtle Dove: Dietary associations with body condition and competition with other columbids analysed using high‐throughput sequencing. Mol Ecol 27:3386–3407. https://doi.org/10.1111/mec.14766

Glutz von Blotzheim UN, Bauer KM (1987) Handbuch der Vögel Mitteleuropas. Band 9. Columbiformes — Piciformes. AULA-Verlag, Wiesbaden.

Gutiérrez-Galán A, Alonso C (2016) European Turtle Dove *Streptopelia turtur* diet composition in Southern Spain: the role of wild seeds in Mediterranean forest areas. Bird Study 63:490–499. https://doi.org/10.1080/00063657.2016.1236070.

Jimenez R, Hodar JA, Camacho I (1992) La alimentación estival de la tórtola común (*Streptopelia turtur*) en el sur de España. Gibier Faune Sauvage 9:119–126.

Kiss JB, Rekasi J, Sterbetz I (1978) Dati Sull’alimentazione di alcune specie di uccelli nel nord della Dobrugia (Romania). Avocetta 2:3–18.

Mansouri I, Al-Sadoon MK, Rochdi M, Paray BA, Dakki M, Elghadraoui L (2019) Diversity of feeding habitats and diet composition in the turtle doves *Streptopelia turtur* to buffer loss and modification of natural habitats during breeding season. Saudi J Biol Sci 26:957–962. https://doi.org/10.1016/j.sjbs.2018.11.006

Murton RK, Westwood NJ, Isaacson A (1964) The feeding habits of the woodpigeon *Columba palumbus*, Stock Dove *C. oenas* and Turtle Dove *Streptopelia turtur*. Ibis 106:174–188.

Murton RK, Westwood NJ, Isaacson AJ (1965) Russian observations by G. N. Likhachev on the diet of the Stock Dove *Columbas oenas* and Turtle Dove *Streptopelia turtur*. Ibis 107:254–256.

**Table S7**. Stock Dove *Columba oenas* diet composition: Compilation of our results and results from previous studies. Given are the plant families and animal prey taxa found in the diet of Stock Doves based on different methods. The plant or animal item is marked with “x” if it was found in the respective study

|  | Present study | Dunn et al. 2018 | Möckel 1988^a^ | Möckel 1988^b^ | Murton et al. 1965^c^ | Murton et al. 1964 | Niethammer & Przygodda 1954 |
| --- | --- | --- | --- | --- | --- | --- | --- |
| **Method** | molecular  (faeces) | molecular  (faeces) | visual  (crop + stomach) | visual  (stomach) | visual  (crop + gizzard) | visual  (crop) | visual  (crop + stomach) |
| **Sampling country** | DE | UK | DE | RO | RU | UK | DE |
| **Sampling years** | 2013-19 | 2011-14 | 1946-63 | NA | 1937-39 | 1958-60 | 1953 |
| **Sample size** | n = 71 | n = 13 | n = 46 | n = 22 | n = 85 | n = 180 | n = 7 |
| Plant family |  |  |  |  |  |  |  |
| Amaranthaceae | x | x |  | x |  | x |  |
| Apiaceae |  | x |  |  |  |  |  |
| Araliaceae | x |  |  |  |  |  |  |
| Asteraceae | x | x | x | x |  |  |  |
| Balsaminaceae | x |  |  |  |  |  |  |
| Boraginaceae |  | x |  |  |  |  |  |
| Brassicaceae | x | x | x | x |  | x | x |
| Cannabaceae | x | x |  |  |  |  |  |
| Caryophyllaceae | x | x | x |  | x | x |  |
| Cucurbitaceae | x |  |  |  |  |  |  |
| Cyperaceae | x |  |  | x |  |  |  |
| Elaeagnaceae | x |  |  |  |  |  |  |
| Euphorbiaceae | x |  |  |  | x | x |  |
| Fabaceae | x | x | x | x | x | x | x |
| Fagaceae | x | x |  |  |  |  |  |
| Geraniaceae |  | x |  |  |  |  |  |
| Juglandaceae | x |  |  |  |  |  |  |
| Lamiaceae |  |  |  |  |  |  | x |
| Onagraceae | x |  |  |  |  |  |  |
| Pinaceae | x |  |  |  |  |  |  |
| Plantaginaceae | x | x |  |  |  | x |  |
| Poaceae | x | x | x | x | x | x | x |
| Polygonaceae |  |  | x | x | x | x |  |
| Primulaceae |  | x |  |  |  | x |  |
| Ranunculaceae |  | x | x |  |  |  |  |
| Resedaceae |  | x |  |  |  |  |  |
| Rosaceae | x | x | x |  |  |  |  |
| Rubiaceae |  |  |  |  |  | x | x |
| Sapindaceae | x |  |  |  |  |  |  |
| Solanaceae | x |  |  |  |  |  |  |
| Urticaceae | x | x |  |  |  |  |  |
| Violaceae |  | x |  |  |  | x |  |
|  | Present study | Dunn et al. 2018 | Möckel 1988 | Möckel 1988 | Murton et al. 1965 | Murton et al. 1964 | Niethammer & Przygodda 1954 |
| **No. families** | 22 | 18 | 8 | 7 | 5 | 11 | 5 |
|  |  |  |  |  |  |  |  |
| Animal prey |  | Not analysed |  | Not mentioned |  |  |  |
| Gastropoda |  | - | x | - | x | x | x |
| Bivalvia |  | - | x | - | x |  |  |
| Clitellata | x | - |  | - |  | x |  |

^a^ only nestlings were sampled. Data from Gasow H. (1977) Über die Ansiedlung der Hohltaube und die Nahrung der älteren Nestlinge von Hohltaube und Ringeltaube.
 Mitt. Landesamt Ökol. Landschaftsentw. Forstplan. Nordrhein-Westf. 3: 207-213 and 277-282

^b^ information from Scherner E.R. (1980) Columba oenas Linnaeus 1758 – Hohltaube. In: Handbuch der Vögel Mitteleuropas 9: 42-64

^c^ data from a Russian paper by Likhachev G.N. (1954) Byulleten ILI. 0-va Isp. Prirodi Otd. Biol. T. 15-25. Percentages of less than 10 were not included

References:

Dunn JC, Stockdale JE, Moorhouse-Gann RJ, et al. (2018) The decline of the Turtle Dove: Dietary associations with body condition and competition with other columbids analysed using high‐throughput sequencing. Mol Ecol 27:3386–3407. https://doi.org/10.1111/mec.14766.

Möckel R (1988) Die Hohltaube: *Columba oenas*. Die neue Brehm-Bücherei, Wittenberg Lutherstadt: Ziemsen.

Murton RK, Westwood NJ, Isaacson A (1964) The feeding habits of the woodpigeon *Columba palumbus*, Stock Dove *C. oenas* and Turtle Dove *Streptopelia turtur*. Ibis 106:174–188.

Murton RK, Westwood NJ, Isaacson AJ (1965) Russian observations by G. N. Likhachev on the diet of the Stock Dove *Columbas oenas* and Turtle Dove *Streptopelia turtur*. Ibis 107:254–256.

Niethammer G, Przygodda W (1954) Zur Ernährung von Ringel- und Hohltaube. Beobachtungen an einem Schlafplatz bei Bonn. Die Vogelwelt 75:41–55.

**Table S8.** Recommended sown mixture for the creation of a Turtle Dove *Streptopelia turtur* foraging site according to the agri-environmental scheme called ‘Turteltauben Brache’ in Hesse, Germany in the framework of the hessian HALM-programme (**H**essian programme for **A**gri-environmental and **L**andscape Management **M**easures). The recommended seeding rate is 5 kg/ha, however, these should be adapted to different soil types. The intended aim is to achieve a patchy, low-growth, early-seeding, seed-rich wild plants foraging site with vegetation-free, i.e. open, soil areas. The vegetation height should not significantly exceed 12 cm in the period from April to August. Ground cover should be a maximum of 50%.

| **Proportion [%]** | **Plant species latin name** | **Plant species common name** |
| --- | --- | --- |
| 15 | *Lotus corniculatus* | common bird's-foot trefoil |
| 15 | *Medicago lupulina* | black medick |
| 15 | *Echium vulgare* | blueweed |
| 10 | *Anthriscus sylvestris* | cow parsley |
| 6 | *Trifolium pratense* | red clover |
| 6 | *Dactylis glomerata* | cock’s-foot |
| 6 | *Geranium dissectum* | cut-leaved crane's-bill |
| 5 | *Fumaria officinalis* | fumitory |
| 5 | *Anagallis arvensis* | scarlet pimpernel |
| 5 | *Viola arvensis* | field pansy |
| 5 | *Plantago major* | broadleaf plantain |
| 3 | *Crepis capillaris* | smooth hawksbeard |
| 3 | *Lythrum salicaria* | purple loosestrife |
| 1 | *Bellis perennis* | common daisy |


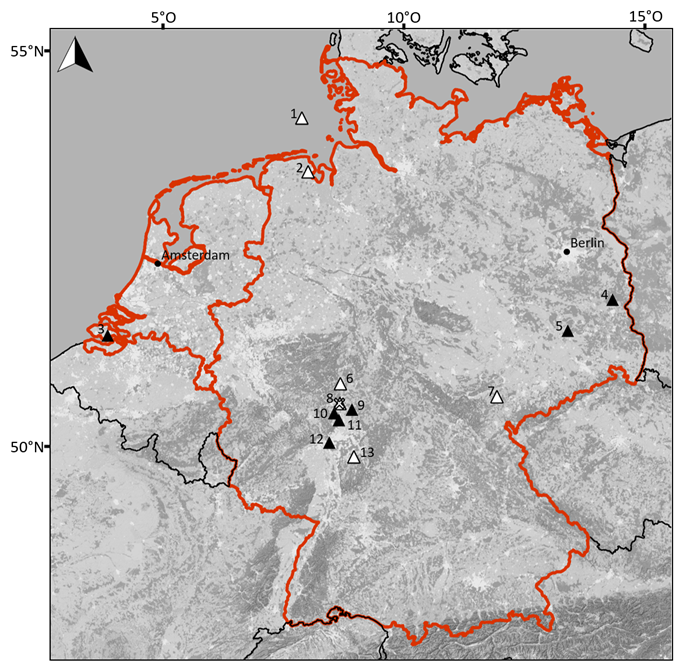


**Figure S1**. Sampling locations of faecal samples of three species from the order of Columbiformes (Common Woodpigeon *Columba palumbus*, European Turtle Dove *Streptopelia turtur*, Stock Dove *C. oenas*) in Germany and the Netherlands. Black triangles represent temporarily baited sites and white triangles sites without bait. 1: Helgoland, 2: Wilhelmshaven, 3: Zak van Zuid-Beveland, 4: Lieberoser Heide, 5: Lausitz, 6: Caldern, 7: Zeulenroda, 8: Giessen, 9: Hungen-Villingen, 10: Cleeberg, 11: Eichkopf, 12: Weilbacher Kiesgruben, 13: Groß-Umstadt. The cross marks the location of the clinic for birds (‘Vetmed’, WP). For exact sample numbers per site see Table S1


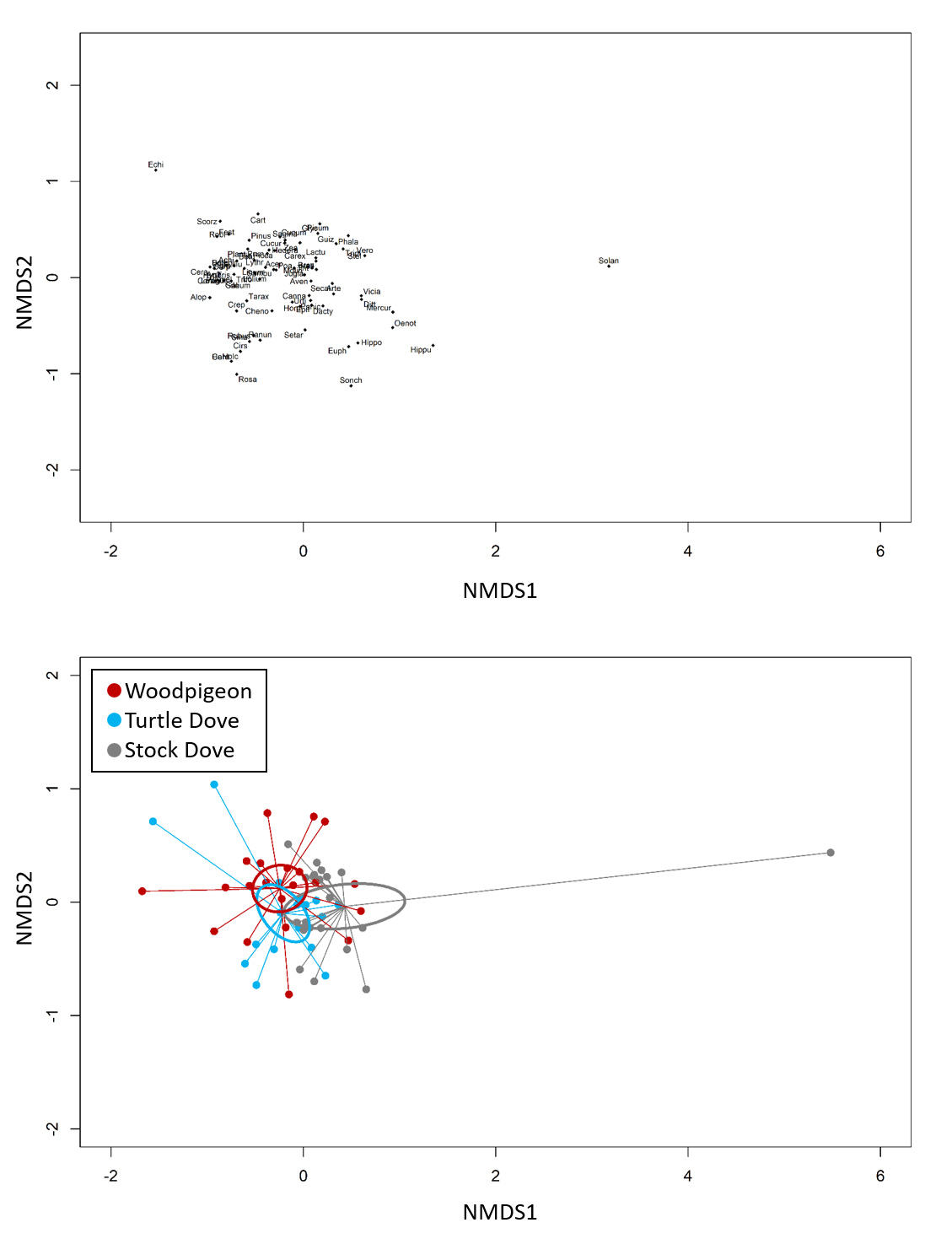


**Figure S2**. Differences in the diet composition at plant genus level in three columbiform species (Common Woodpigeon *Columba palumbus* (WP); European Turtle Dove *Streptopelia turtur* (TD); Stock Dove *C. oenas* (SD)), using Non-metric Multidimensional Scaling (NMDS, function *metaMDS* in the R package ‘VEGAN’). Depicted are (A) the distribution of the plant genera (the first four or five letters of the genera are given) and (B) the distribution of samples and 95% confidence ellipses
